# Exploratory study of cold hypersensitivity in Japanese women: genetic associations and somatic symptom burden

**DOI:** 10.1038/s41598-024-52119-y

**Published:** 2024-01-22

**Authors:** Xuefeng Wu, Tetsuhiro Yoshino, Ayako Maeda-Minami, Sachiko Ishida, Masami Tanaka, Akinori Nishi, Yoshio Tahara, Ryohei Inami, Aiko Sugiyama, Yuko Horiba, Kenji Watanabe, Masaru Mimura

**Affiliations:** 1 Center for Kampo Medicine, Keio University School of Medicine, Tokyo, 160-8582 Japan; 2 Holistic Kampo Diagnosis Laboratory, Keio University School of Medicine, Tokyo, 160-8582 Japan; 3 Faculty of Pharmaceutical Sciences, Tokyo University of Science, Chiba, 278-0022 Japan; 4 DeNA Life Science Inc., Tokyo, 150-6140 Japan; 5 TSUMURA Advanced Technology Research Laboratories, TSUMURA & CO., Ibaraki, 300-1192 Japan; 6 Department of Neuropsychiatry, Keio University School of Medicine, Tokyo, 160-8582 Japan

**Keywords:** Genetics, Clinical genetics, Genetic association study, Medical genetics, Clinical genetics, Chronic pain

## Abstract

Temperature perception is essential for humans to discern the environment and maintain homeostasis. However, some individuals experience cold hypersensitivity, characterized by a subjective feeling of coldness despite ambient environmental temperatures being normal, the underlying mechanisms of which are unknown. In this study, we aimed to investigate the relationship between subjective cold symptoms and somatic burden or single nucleotide polymorphisms to understand the causes of cold hypersensitivity. We conducted an online questionnaire survey [comprising 30 questions, including past medical history, subjective symptoms of cold hypersensitivity, and the Somatic Symptom Scale-8 (SSS-8)]. Respondents were 1200 Japanese adult female volunteers (age: 20–59 years), recruited between April 21 and May 25, 2022, who were customers of MYCODE, a personal genome service in Japan. Among the 1111 participants, 599 (54%) reported cold hypersensitivity. Higher cold hypersensitivity severity was positively associated with the SSS-8 scores. Additionally, a genome-wide association study for cold hypersensitivity was conducted using array-based genomic data obtained from genetic testing. We identified 11 lead variants showing suggestive associations (*P *< 1 × 10^–5^) with cold hypersensitivity, some of which showed a reasonable change in expression in specific tissues in the Genotype-Tissue Expression database. The study findings shed light on the underlying causes of cold hypersensitivity.

## Introduction

Normal cold sensation is essential for humans to perceive the surrounding environment and thermoregulation. However, some individuals report experiencing cold hypersensitivity (Hie or Hiesho in Japanese), characterized by a subjective cold sensation over the body despite the ambient temperature being within the normal range^1,2^. Individuals with cold hypersensitivity may experience delayed rewarming due to prolonged vasoconstriction when exposed to cold environments or sudden temperature drops^3–5^.

Additionally, cold hypersensitivity can cause distress and hinder the execution of routine activities^6^. In Japan, women with cold hypersensitivity report a painful cold sensation and associated symptoms such as insomnia, fatigue, and edema^7^. Further, cold hypersensitivity is associated with developing chronic conditions, such as dysmenorrhea, rheumatic diseases, migraines, and vascular diseases^4,8^. Therefore, understanding the etiology and pathophysiology of cold hypersensitivity is essential for effective management and treatment.

The underlying cause of cold hypersensitivity remains unclear. Several possible physiological mechanisms have been suggested, including menopausal vasomotor dysregulation because of female estrogen depletion^9^ and decreased heat production because of reduced muscle mass in sarcopenia or hypothyroidism^10^. However, these underlying conditions are absent in many individuals experiencing cold hypersensitivity.

Psychological factors are another possible cause of cold hypersensitivity. Physical coldness is an embodied cognition related to high anxiety sensitivity^11^, social exclusion^12,13^, and money reminders^14^. We found that individuals with cold hypersensitivity tended to have more accompanying symptoms than normal individuals^15^. While the number of physical symptoms has a predictive value for mental disorders with somatization^16^, no research has yet surveyed the somatic burden in the cold hypersensitivity group using standardized psychological scores, such as the Patient Health Questionnaire somatic symptom severity scale (PHQ-15)^17^ or its short version, the Somatic symptom scale-8 (SSS-8)^18^.

Considering cold sensitivity is a highly subjective perception, an alternative explanation is that it is caused by dysfunctional temperature sensation, which may be genetically determined by previous epidemiological studies^19,20^. Additionally, we previously reported a constant prevalence of cold hypersensitivity from puberty onward, suggesting the importance of a congenital factor rather than the acquired one^15^. The frequency of rs10166942, a single nucleotide polymorphism (SNP), in transient receptor potential cation channel subfamily melastatin member 8 (*TRPM8*), a cold sensor, increased with increasing latitude, suggesting that *TRPM8*-mediated cold perception is associated with human adaptation to cold environments^21^. Furthermore, recent genetic analyses have provided insights into a range of candidate genes and variants associated with cold hypersensitivity. Studies have also identified specific genes related to thyroid hormone regulation^22,23^ and the neuro-endocrine-immune system^24^ potentially linked to cold syndrome. Additionally, variations in β-adrenergic receptors, uncoupling protein 1, and angiotensinogen have been implicated in cold constitution^25^. Notably, research has improved our understanding of the role of *TRPM8* in cold sensation and pain sensitivity, particularly highlighting the association of the SNP rs12992084 with cold pain sensitivity^26^. These findings, while preliminary and often based on small sample sizes, underscore the complexity and genetic heterogeneity of cold hypersensitivity.

A genome-wide association study (GWAS) is a standard genetic approach for exploring the potential susceptibility of genetic variants for a particular trait or disease^27,28^. However, no GWAS has assessed cold hypersensitivity. In this study, we conducted an online survey to assess the relationship between subjective cold symptoms and somatic burden. To uncover the root cause of cold hypersensitivity, we employed a GWAS approach to identify genetic variants. The survey was conducted with adult Japanese female volunteers who had already undergone MYCODE genetic testing at the time of the survey.

## Methods

### Study design and sample size calculation

We calculated the sample size to be 1100 to meet a genome-wide significance level of ${\textit{P}} \le 5\times {10}^{-8}$ with a power of 0.8 in an additive model, considering a proportion of 0.7 for cold hypersensitivity^15^, a relative risk ratio of 1.2, and a minor allele frequency of 0.4. Additional 100 participants were included to account for potential dropouts and withdrawals. Participation was voluntary, and all participants provided informed consent for this study. This study adhered to the principles outlined in the Declaration of Helsinki. The research protocol was approved by the Keio University School of Medicine Ethics Committee (approval number: 20211133). The protocol is available at the UMIN clinical trials registry (unique ID: UMIN000047040).

### Inclusion and exclusion criteria

From customers of MYCODE (DeNA Life Science, Inc., Tokyo, Japan), a personal genome service in Japan, 1200 Japanese adult female volunteers (aged 20–59 years) were recruited between April 21 and May 25, 2022. Customers who provided consent for MYCODE research, in which their anonymized genetic data and health-related information would be used for scientific research purposes, were invited to participate in this study via email from DeNA Life Science.

All participants completed an online survey of 30 questions, including attribute information, current medical history, subjective symptoms of cold hypersensitivity, and SSS-8 (Supplementary Table 1). Our exclusion criteria included: discrepancies in sex or age compared to their previous registration information; age less than 20 years or above 59 years; diagnosis of heart failure, abnormal thyroid function (hyperthyroidism, hypothyroidism, chronic thyroiditis, or Hashimoto’s disease), or malignancy; hospitalization at the time of the survey; pregnant or potentially pregnant; and extreme responses for age/height/weight judged by the authors.

We excluded 65 participants: two participants aged under 20 or over 59 years, four males, 21 participants currently diagnosed with exclusion diagnoses, 29 participants hospitalized at the time of the survey, three participants that were pregnant or potentially pregnant, five participants showing discrepancies in age between the answer and the MYCODE registration information, and one participant with an unrealistic BMI value (over $100 {\text{kg}}/{{\text{m}}}^{2}$).

### Cold hypersensitivity assessment

The cold hypersensitivity assessment was revised from the Japanese questionnaire used in the Kampo Clinic at Keio University Hospital^7,29^. The questionnaire covers topics such as lifestyle, presence of cold sensitivity, location (extremities, lower body, full body), severity (rank 1–5), and age of onset (10s, 20s, 30s, 40s, 50s). Because up to 30% of individuals with cold hypersensitivity experience hot flashes or heat sensations in the face^15^, the questionnaire includes questions about the presence and severity of hot flashes.

### Somatic symptom burden assessment

The somatic symptom burden was measured using SSS-8^18,30^, which consists of 8 rating scale items scored from 0 (‘not at all’) to 4 (‘very much’). Severity categories were determined by the sum of the scores: 8–11, medium; 12–15, high; and 16–32, very high.

### Sample quality control

Subsequently, 24 of the remaining 1135 participants were further removed through stringent quality control procedures based on genotyping and imputed data using PLINK (ver 1.9): three participants with discordant sex, five participants with a call rate of less than 0.98, seven participants who were one individual of a pair with identity by descent (PI_HAT) over 0.1875, and nine participants identified as outliers from the Japanese cluster based on genetic principal component analysis (PCA). First, we performed PCA on the genotype data of participants using East Asian samples (n = 504) from the 1000 Genomes Project Phase 3 (1KGP, n = 2504). We excluded seven outliers from the Japanese cluster over the threshold determined by visual inspection of the first two principal components (PCs) (Supplementary Fig. 1A). Second, PCA was performed on the data of the studied samples, and two apparent outliers were removed by visual inspection (Supplementary Fig. 1B–D). The final dataset included 1111 participants.

### Genotyping and SNP quality control

Before being recruited for this study, participants completed MYCODE genetic testing by providing saliva samples to the DeNA Life Science laboratory. An Infinium OmniExpress-24 or Human OmniExpress-24 BeadChip (Illumina Inc., San Diego, CA, United States) containing approximately 30,000 custom probes was used. SNP coordinates were annotated in the GRCh37 genome. We performed SNP quality control on 684,428 biallelic, autosomal variants. We excluded SNPs with call rate < 0.99, Hardy–Weinberg equilibrium (${\textit{P}}<1.0\times {10}^{-6}$), minor allele frequency (MAF) < 0.01, over 0.1 allele frequency difference with the ToMMo 4.7KJPN-SNV/INDEL Allele Frequency Panel (4.7KJPN) obtained from the Japanese Multi Omics Reference Panel (jMorp). All the abovementioned procedures were performed using PLINK (ver 1.9)^31,32^.

### Genotype imputation

Genotype imputation was performed on 492,418 autosomal variants after SNP quality control using Eagle (version 2.4.1)^33,34^ for the phasing step and Minimac3 (version 2.0.1)^35^ for imputation using all 1KGP samples. Post-imputation quality control was performed on 43,835,815 variants. We excluded variants with low imputation quality (${r}^{2}$ < 0.7), multi-allelic SNPs, and those not observed in 4.7KJPN as biallelic SNPs or those with > 0.1 allele frequency difference with 4.7KJPN. In each association test, SNPs with call rates < 0.99, MAF < 0.05, and Hardy–Weinberg equilibrium (${\textit{P}}<1.0\times {10}^{-6}$) were excluded.

### Trait correlation analyses

Correlation analysis between 12 traits (including 9 items related to cold sensitivity and 3 items related to hot flashes) and eight attribute factors of demographic information and medication history was conducted using Spearman’s rank correlation test (cor.test) with the statistical analysis software R Version 4.1.1^36^. Hierarchical clustering was performed using the Euclidean distance from Ward’s method using hclust. A heat map was drawn using heatmap.2 from the gplols package in R (Supplementary Fig. 2). The significance *P* value threshold by Bonferroni correction was $0.05/96=5.2\times {10}^{-4}$ where 96 is the number of combinations of 12 traits and 8 factors.

### Association studies

GWASs were conducted on each cold sensitivity trait using logistic regressions implemented in PLINK (ver 1.9)^31,32^. In each GWAS, we kept the control group constant, consisting of 512 participants without cold hypersensitivity among the 1111 individuals in the study. For binary traits, we labeled “Yes’’ answers as cases. For categorical traits, such as cold severity scale and age of onset, we converted them into binary variables (see Supplementary Table 2). The number of case groups and SNPs analyzed for each trait can be found in Supplementary Table 2.

Cold hypersensitivity is a pathological condition in Kampo medicine and a frequent subjective complaint in Kampo clinics^15^. Kampo treatment can be obtained from a drug store or prescribed by a doctor, making it challenging to determine whether it targets cold hypersensitivity because treatment principles are based on pattern diagnosis, which considers the subjective complaint and accompanying symptoms^7^. To minimize potential Kampo treatment influence, we performed a substratification analysis on 907 subjects who did not consume Kampo medicines daily (see Supplementary Table 3).

We applied an additive model (the SNP effect on the phenotype was proportional to the allele number) and used age, BMI, menopausal status, and PCs 1–5 from the PCA of SNP data as covariates in logistic regression. We set the genome-wide significance level at (${\textit{P}}<5\times {10}^{-8}$) and the suggestive significance level at (${\textit{P}}<1\times {10}^{-5}$). We determined the lead SNPs independently of each other using the clump option (${r}^{2}$ < 0.2, 10 Mbp window) in PLINK.

We visualized the association test results using the qqman package in R and created QQ and Manhattan plots. Moreover, we created detailed regional plots of the associations using LocusZoom and the 1KGP Asian population (ASN, March 2014) data as reference panels for linkage disequilibrium (LD). Finally, we used SNPnexus for functional annotation of the identified related SNPs based on reference genome build GRCh37.

### eQTL search using GTEx release V8

To investigate the effects of the identified SNPs on gene expression in each tissue, we searched for cis-expression quantitative trait loci (﻿eQTLs) in the Genotype-Tissue Expression (GTEx) Analysis Release V8 database. The significance of cis-eQTLs was based on the Q-value threshold in the GTEx database.

### Statistical analysis

R software was used for all statistical analyses. Continuous and categorical variables were compared across cold sensitivity status using Mann–Whitney’s U and Pearson’s chi-square tests, respectively. The Jonckheere–Terpstra trend test was performed to examine the association between cold hypersensitivity severity and SSS-8 scores. ${\textit{P}}<0.05$ was considered statistically significant. *P* values for pairwise differences between groups were calculated using the Holm–Bonferroni method.

## Results

### Participants

**Figure 1:**
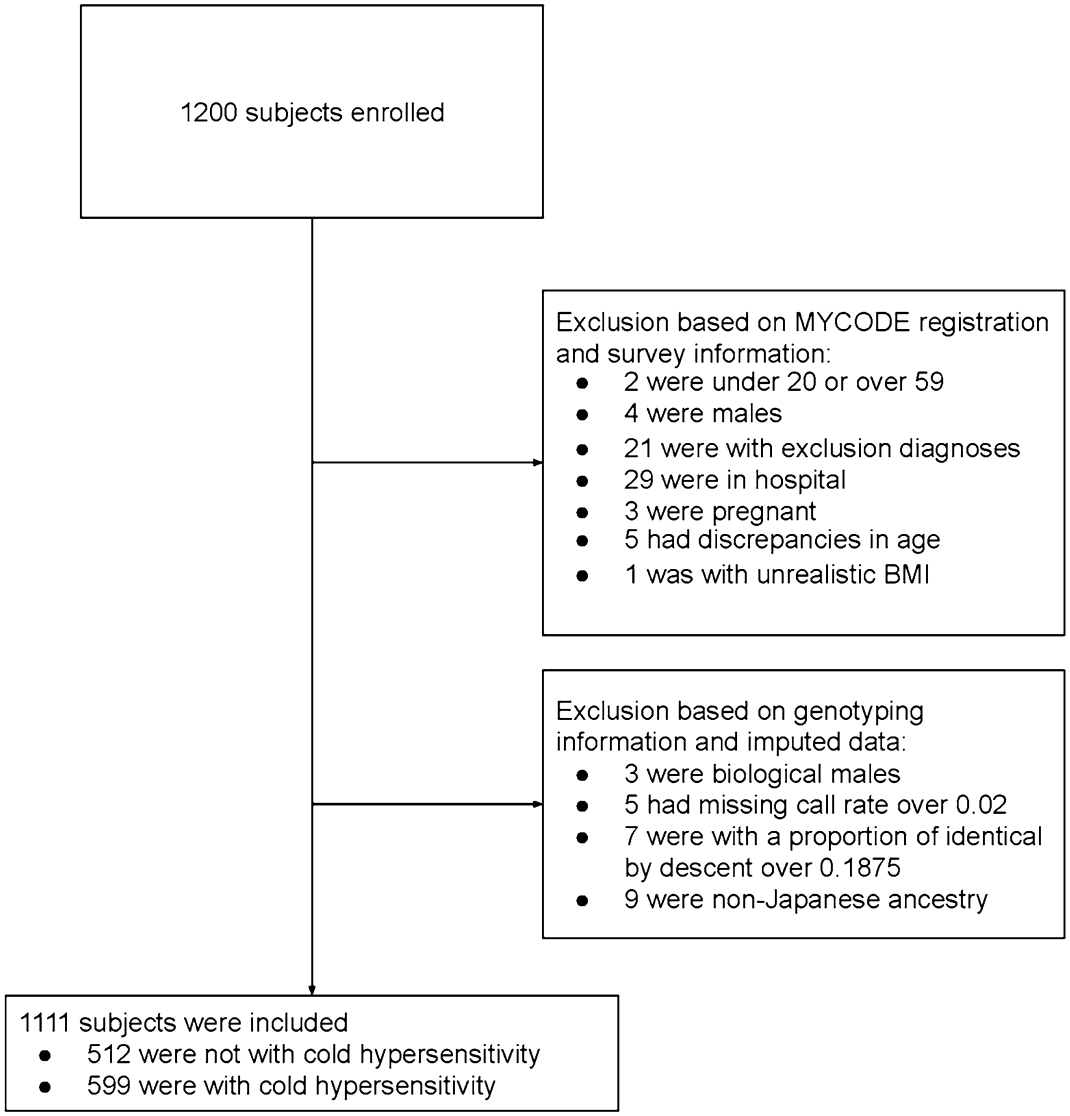
Subjects flow chart.

A total of 1200 subjects were enrolled in the study, of whom 1111 met the inclusion criteria (Fig. 1). Of these 1111 participants, 599 (54%) reported cold hypersensitivity. The demographic characteristics of the participants are shown in Table 1, and they demonstrate that participants with cold hypersensitivity tend to have smaller body mass indexes (BMIs) (20.7 ${\text{kg}}/{{\text{m}}}^{2}$ vs. 21.3 ${\text{kg}}/{{\text{m}}}^{2}$) and smaller body weights (52.2 ${\text{kg}}$ vs. 54.0 ${\text{kg}}$) compared to those of normal individuals. They were also less likely to be menopausal (27% vs. 29%), less likely to participate in sports (26% vs. 34%), more likely to complain of hot flashes (28% vs. 14%), have higher median SSS-8 scores (9 vs. 6), and were more likely to receive Japanese traditional herbal treatments (Kampo medicines) (22% vs. 14%).

**Table 1:** Demographic backgrounds.

	Cold sensitivity status			*P* value
	Overall (n = 1111)	Normal (n = 512)	Hypersensitivity (n = 599)	
Age, years, median (range)	47 (20–59)	47 (20–59)	46 (20–59)	0.157
BMI, kg/m^2^, median (range)	21 (15.1–49.4)	21.3 (15.3–49.4)	20.7(15.1–39.5)	< 0.001
Height, cm, median (range)	159 (143–175)	159 (145–175)	159 (143–175)	0.667
Weight, kg, median (range)	53.0 (34.5–125.0)	54.0 (36.0–125.0)	52.2 (34.5–108.0)	0.003
Menopaused,* n* (%)	315 (28)	151 (29)	164 (27)	< 0.001
Regular sports habit, *n* (%)	332 (30)	176 (34)	156 (26)	0.002
Skipping breakfasts,* n* (%)	394 (35)	171 (33)	223 (37)	0.183
Walk speed, *n* (%)				
Slow	113 (10)	43 (8)	70 (12)	0.066
Normal	567 (51)	255 (50)	312 (52)	
Fast	431 (39)	214 (42)	217 (36)	
Hot flashes, *n* (%)	243 (22)	74 (14)	169 (28)	< 0.001
SSS-8 scores, median (range)	8 (0–30)	6 (0–30)	9 (0–26)	< 0.001
Participants with comorbidities,* n* (%)	392 (35)	176 (34)	216 (36)	0.558
Medications				
Kampo medicines, *n* (%)	204 (18)	74 (14)	130 (22)	0.002
Anti-hypertensives,* n* (%)	40 (4)	20 (4)	20 (3)	0.613
HRT, *n* (%)	119 (11)	45 (9)	74 (12)	0.055
Vasodilators, *n* (%)	13 (1)	6 (1)	7 (1)	0.996

Continuous and categorical variables were compared across cold sensitivity status using Mann–Whitney's U, and Pearson's Chi-square tests, respectively.

*BMI* body mass index, *HRT* hormone replacement therapy, *SSS-8* somatic symptom scale-8.

Of the 599 participants with cold hypersensitivity, 207 (34.6%) claimed that their symptoms started between 10 and 19 years (Supplementary Table 4), suggesting that cold hypersensitivity is a common symptom appearing from a young age. In addition, 233 (38.9%) participants indicated that they were "quite a bit" or "very much" bothered by cold hypersensitivity.

Correlation analyses revealed several significant correlations between BMI, age, menopause condition, regular hospital visits, medication status, cold hypersensitivity, and hot flash symptoms. However, the correlation coefficients were less than 0.2 (Supplementary Fig. 2).

### Association between cold hypersensitivity and SSS-8 score

**Figure 2:**
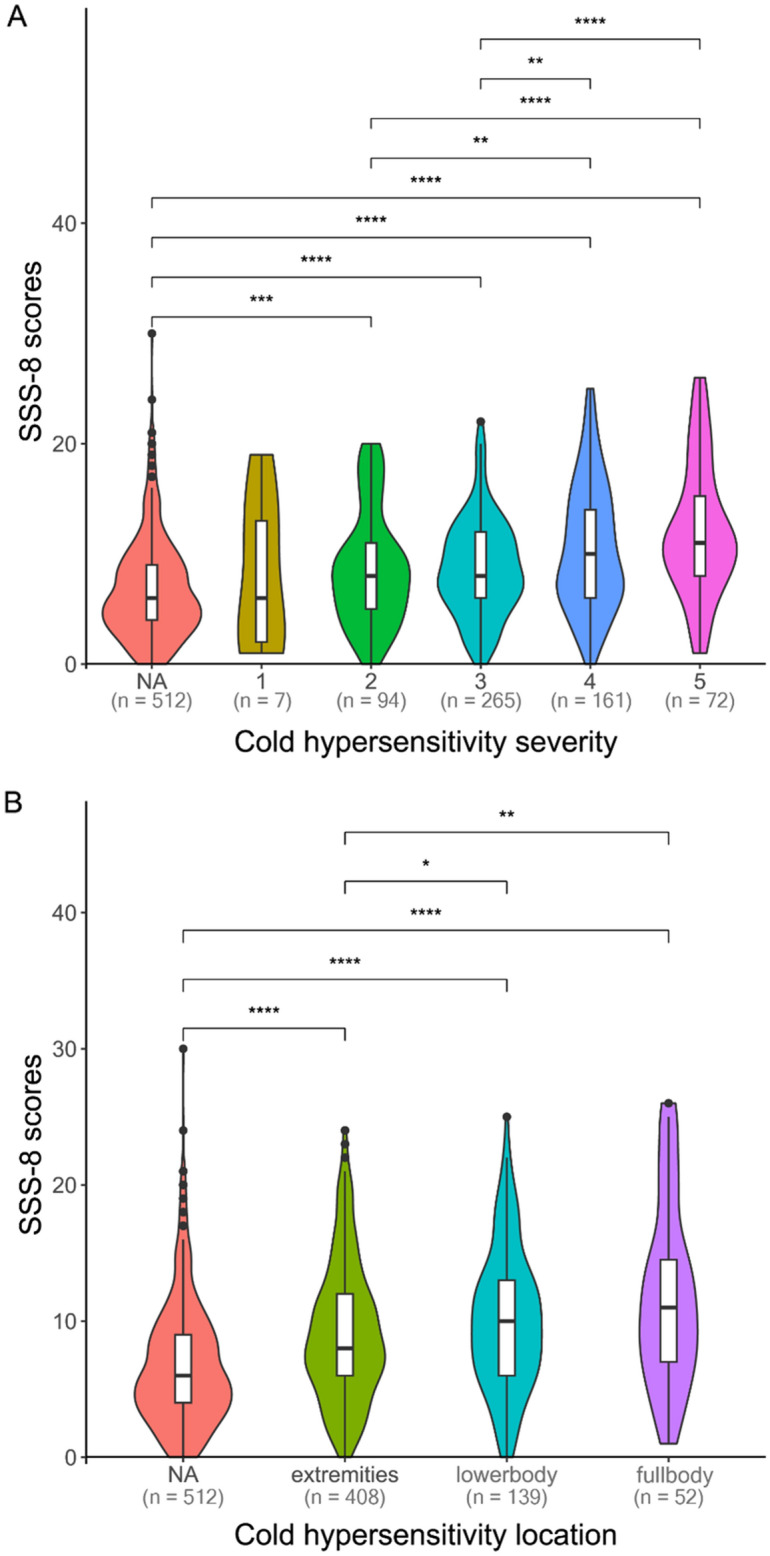
Associations of the somatic symptom scale-8 (SSS-8) scores with (**A**) cold hypersensitivity severity and (**B**) location. The x-axis in (**A**) represents severity, with values ranging from 1 (not at all) to 5 (very much). *NA* not applicable for cold hypersensitivity severity or location, i.e., the population with normal cold sensitivity status (n = 512). *P *values for pairwise differences between groups were calculated using the Holm–Bonferroni method. $$^{*}{\textit{P}}\le 0.05; ^{**}{\textit{P}}\le 0.01; ^{***}{\textit{P}}\le 0.001; ^{****}{\textit{P}}\le 0.0001$$.

We examined the relationship between cold hypersensitivity and SSS-8 scores. Figure 2A shows that higher cold hypersensitivity severity was associated with higher SSS-8 scores, substantiated by the Jonckheere–Terpstra trend test (${\textit{P}}<2.20\times {10}^{-16}$). Figure 2B illustrates the relationship between cold hypersensitivity locations and SSS-8 scores. We found that participants with cold hypersensitivity in the extremities (n = 408) had lower SSS-8 scores than those with lower-body (n = 139, *P* = 0.043) or full-body cold hypersensitivity (n = 52, *P* = 0.007).

### SNPs suggestively associated with cold hypersensitivity

To discern the genetic factors influencing cold hypersensitivity, we carried out a series of GWAS, using logistic regression analysis with adjustments for basic and relevant clinical factors as covariates (age, BMI, menopausal status, and SNP-derived principal components 1 through 5).

**Figure 3:**
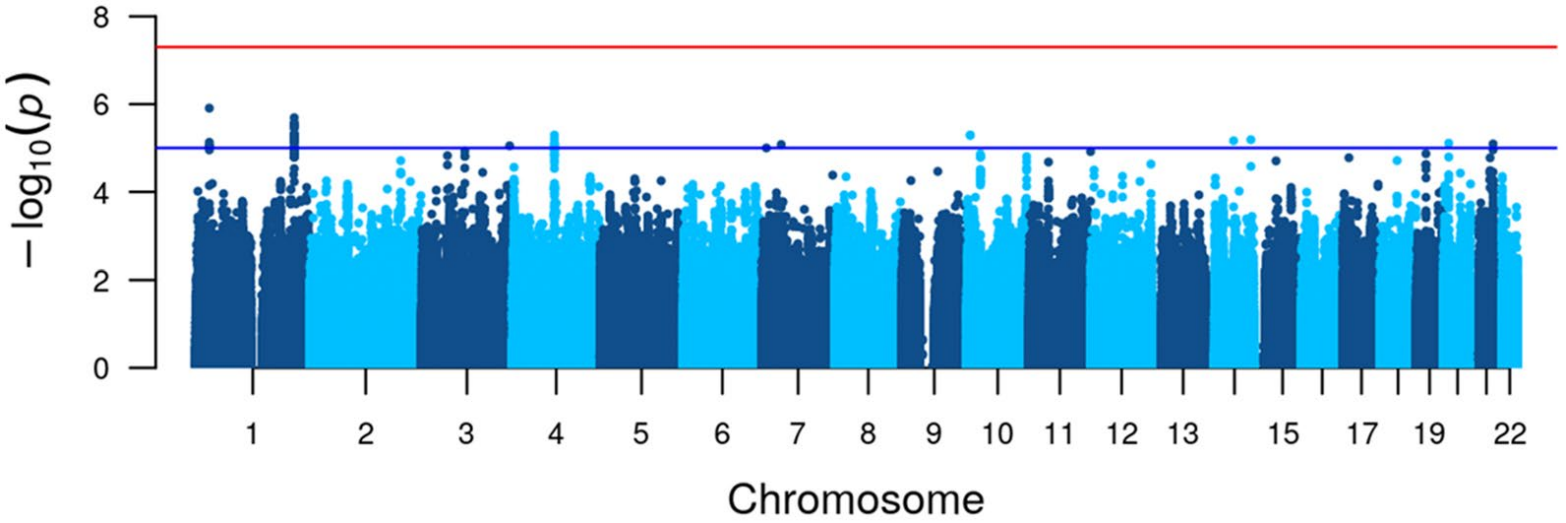
Manhattan plot for genetic associations with the presence of cold hypersensitivity. The red line indicates the genome-wide significance threshold $5\times {10}^{-8}$. The blue line indicates the suggestive significance threshold $1\times {10}^{-5}$.

Our primary analysis aimed to determine the relationship between genetic diversity and cold hypersensitivity. Although no associations surpassing the genome-wide significance threshold were found (${\textit{P}}<5\times {10}^{-8}$, Fig. 3), 11 variants showed genome-wide suggestive associations (${\textit{P}}<1\times {10}^{-5}$, Table 2) with the presence of cold hypersensitivity. In a series of sensitivity analyses to ensure the robustness of these results, we further explored cold hypersensitivity via related subordinate traits such as location, severity, and onset stratified by life’s decade (Supplementary Figs. 3, 4, 5 and Supplementary Table 5). We focused on gene regions previously associated with temperature sensation and regulation in animal studies^37,38^, namely potassium two-pore domain channel subfamily K member 2 (*KCNK2*) and *TRPM2*.

**Table 2:** GWAS-identified SNPs and corresponding genes via eQTL search in cold hypersensitivity.

SNP	CHR	BP	A1	A2	MAF	*P* value	Odds ratio	SE	eGenes
rs750707	1	30,282,320	G	A	0.42	1.24E−06	1.56	0.09	–
rs1869201	1	215,173,014	G	A	0.42	2.04E−06	0.66	0.09	*KCNK2*
rs62295776	3	191,853,763	A	T	0.27	8.91E−06	0.64	0.10	–
rs10016576	4	91,484,515	G	C	0.48	5.07E−06	1.50	0.09	–
rs74513903	7	10,410,139	T	C	0.20	9.98E−06	0.62	0.11	–
rs2656514	7	42,777,914	G	A	0.30	8.35E−06	0.65	0.10	*GLI3*
rs12761019	10	7,788,209	A	G	0.11	5.10E−06	0.52	0.14	*ITIH2, KIN*
rs17098278	14	61,831,860	C	T	0.22	6.85E−06	0.61	0.11	–
rs10220484	14	99,457,631	C	T	0.31	6.49E−06	1.55	0.10	–
rs2876278	20	12,230,024	G	A	0.35	7.78E−06	1.51	0.09	–
rs4818919	21	45,849,864	G	A	0.15	8.09E−06	1.73	0.12	*AP001062.7, TRPM2, MTND5P1, C21orf2*

*SNP* single nucleotide polymorphism, *CHR* chromosome, *BP* base-pair, *GRCh37* genomic coordinate, *A1* minor allele, *A2* major allele, *OR* odds ratio for A1 allele, *SE* standard error of the OR, *MAF* minor allele frequency, *eGenes* expression Quantitative Trait Locus genes, are identified based on a Q-value threshold in the Genotype-Tissue Expression (GTEx) project database.

Specifically, lead SNPs in the regions near the *KCNK2* gene were associated with cold hypersensitivity (Lead SNP rs1869201, ${\textit{P}}=2.04\times {10}^{-6}$, OR = 0.66). The major allele with a frequency of 0.58 was associated with an increased risk of cold hypersensitivity. The signal was found in a region spanning from the 5’ upstream to the anterior region of the *KCNK2* gene with no missense SNPs affecting the amino acid sequence (Supplementary Fig. 4A).

Additionally, we identified SNPs suggestively associated with the presence of cold hypersensitivity near the *TRPM2* gene, which is known as a temperature-sensitive channel (Lead SNP rs4818919, ${\textit{P}}=8.09\times {10}^{-6}$, OR = 1.73). The minor allele with a frequency of 0.15 was associated with an increased risk of cold hypersensitivity (Supplementary Fig. 5A).

Considering our study population included regular users of Kampo medicines, a common treatment for cold hypersensitivity, we analyzed a subset of 907 individuals not frequently using these medicines (Supplementary Table 3). However, the *P* values of the genes we highlighted showed no reduction, suggesting either a limited influence of Kampo medicine on these genes or an effect due to reduced statistical power from the smaller sample size.

As no intolerant non-synonymous SNPs were identified from the GWASs, our attention was directed towards exploring eQTLs in various tissues. In relation to the presence of cold hypersensitivity, we investigated the influence of 11 lead SNPs to examine the effect of SNPs on gene expression. We identified four eQTL hits across nine tissue types in the GTEx database (Table 2, and detailed on Supplementary Table 6). The lead SNP rs1869201 has been associated with reduced *KCNK2* expression in the ovaries when the risk allele A is present (Supplementary Fig. 6). Another lead SNP, rs4818919, has been associated with decreased *TRPM2* expression in brain tissue. However, this SNP demonstrated a reverse effect in peripheral non-brain tissues, such as skeletal muscle and whole blood. The risk allele G for cold hypersensitivity in rs4818919 was associated with increased *TRPM2* expression in these tissues (Supplementary Fig. 7A, B).

## Discussion

This study aimed to investigate the etiology of cold hypersensitivity from psychological and genetic perspectives. Data were collected from 1111 healthy Japanese women (aged 20–59 years) through self-report questionnaires. These women underwent MYCODE genetic testing prior to enrollment. Our study found a cold hypersensitivity prevalence of 53.92%, consistent with other studies reporting 47.78% to 65.68% cold hypersensitivity prevalence in East Asian populations^6,8,9,15^.

We used SSS-8 to assess the somatic burden associated with cold hypersensitivity. The SSS-8 score was positively correlated with the severity of cold hypersensitivity (Fig. 2A). Our study found the median SSS-8 score of nine for the cold hypersensitivity group, comparable with Japanese outpatients with somatic symptoms and related disorders without central sensitization syndrome^39^ and significantly higher than that for the normal group. These results suggest that the somatic burden for cold hypersensitivity is relatively high and possibly similar to that observed in somatic symptom outpatient groups.

Self-reported somatic symptoms can vary across cultures ^40–42^. Matsudaira et al.^30^ reported the mean SSS-8 score for the general Japanese population was 4.5, whereas the mean score for the general German female population was 3.49^18^. Moreover, individuals with cold hypersensitivity tended to seek traditional medicines more often than those in the normal group. Given the higher prevalence of cold hypersensitivity in East Asian populations, cold hypersensitivity may be a common expression of illness perception shared within ancient Chinese culture, with higher rates of somatic symptoms being endorsed rather than psychological symptoms. Therefore, further cross-cultural research is necessary to validate our findings regarding the somatic burden of cold hypersensitivity.

To explore the genetic polymorphism related to cold hypersensitivity, we conducted a GWAS and identified 11 lead variants potentially linked to the main trait, "presence of cold hypersensitivity”. Our study, combined with sensitivity analyses, was the first to identify the *KCNK2* and *TRPM2* regions via research in humans. Despite the novelty of our findings, the GWAS results did not directly correlate with previously reported associated genes or SNPs such as *TRPM8* rs10166942^21^ and rs12992084^26^. This discrepancy in the findings might be partially due to the specific demographics of our cohort, which consisted solely of 1111 adult Japanese women, compared to the more diverse or smaller populations in previous studies. The gender and ethnicity of participants, along with the multifaceted nature of the cold hypersensitivity, can significantly influence genetic association studies, and our focused recruitment might have limited the detection of certain genetic variants widely reported in mixed or smaller cohorts.

Following eQTL searches, it can be suggested that the identified genomic regions regulate the levels of transcripts associated with cold hypersensitivity, rather than exerting a direct influence on protein function. The identification of *KCNK2* and *TRPM2* regions in our study provides new insights into the genetic architecture of cold hypersensitivity.

KCNK2, also known as TREK-1, belongs to the two-pore domain potassium channels (K2P) and is thought to be crucial in cold hypersensitivity. For example, studies using a double knockout animal model of *KCNK2* and *TRAAK*, another K2P channel, reported dorsal root ganglion neuronal hypersensitivity to cold stimuli^37,43^.

Further, the coordinated activity of KCNK2 and TRP channels is involved in temperature detection. Under normal conditions, K2P channels, as background ${K}^{+}$ channels, are activated at a physiological temperature of around 30 °C, driving neuronal hyperpolarization and thus inhibiting neuronal firing. At 10 °C, KCNK2 closes, and the activity of cold-sensitive TRP channels (such as TRPM8) increases, leading to neuronal depolarization and thus facilitating information transmission^44^. The eQTL analysis in our study indicated that the risk allele rs1869201[A] for cold hypersensitivity correlated with lower *KCNK2* expression (Supplementary Fig. 6), suggesting that risk allele carriers tend to exhibit higher cold hypersensitivity owing to lower *KCNK2* expression. We propose that the decreased expression of *KCNK2* causes the neuron to be more easily activated by cold-sensitive TRPM8, which is presumed to be inhibited in warm environments, resulting in the illusion of a cold sensation even in warm environments.

However, the frequency of rs1869201[A] associated with the risk of cold hypersensitivity varies between populations. For example, in the Japanese population, the rs1869201[A] frequency was reported to be 0.59 (8.3 KJPN public data, Japanese Multi Omics Reference Panel). In contrast, in the European population, it was 0.29 as a minor allele (1 KGP CEU). This difference in the frequency of the A allele across populations may explain the difference in cold hypersensitivity prevalence in Japan and Europe.

The TRPM2 channel is a non-selective cation channel activated above 35°C^45^. This channel is crucial in non-noxious warmth detection by peripheral sensory neurons. Additionally, TRPM2, through disinhibition of preoptic warm-sensitive neurons, facilitates deep brain temperature monitoring and body temperature regulation in response to extreme heat (above 39 °C)^38,46–48^. Further, *TRPM2*-knockout mice exhibited a mild preference for a non-noxious warmer environment (38–42 °C) that the wild-type mice avoided ^38^, indicating that a low level of TRPM2 is involved in warm-seeking behavior.

Our eQTL search identified rs4818919 as an eQTL for the *TRPM2* gene, with the effect varying across tissues. The risk allele A corresponded to lower expression in brain tissues (Supplementary Fig. 7A), whereas higher expression was observed in peripheral non-nervous tissues (Supplementary Fig. 7B). Thus, we hypothesize that cold hypersensitivity might be due to a loss of function of warm temperature perception. Specifically, it is plausible that a reduction in *TRPM2* expression within brain tissues may give rise to a sensation of coolness or coldness. In turn, this perception may trigger peripheral vasoconstriction, a physiological response aimed at maintaining core body temperature, thus elucidating the cold sensation complaints in extremities among the majority of individuals with cold hypersensitivity.

Recent research links TRPM2 in the hypothalamus to body temperature reduction during fevers^47,48^ and identifies it as a brain redox sensor^49^. Studies using *TRPM2*-deficient mice have shown behaviors related to bipolar disorder ^50^, possibly explaining the increased somatic burden seen in individuals with cold hypersensitivity. However, the precise role of TRPM2 in thermoregulation and somatic burden requires further study.

Our study acknowledges several limitations. First, thermosensitivity’s subjective nature complicates the accurate diagnosis and measurement of cold sensations, especially when reliant on self-reported, web-based questionnaires. Second, our analyses did not consider geographical, environmental, or socioeconomic factors, including residential temperature, birthplace, occupational cold exposure, familial history, or previous incidents of frostbite, which may considerably influence the perception and prevalence of cold hypersensitivity. Therefore, our analyses may not have fully adjusted for these potentially significant factors. Third, due to the subjective nature of cold sensitivity and the wide range of related traits, we employed a relatively generous threshold to interpret GWAS results. This approach allowed us to identify potential genetic regions of interest; however, it necessitates further validation to fully comprehend the complex interrelationships among cold hypersensitivity, somatic burden, and genetic diversity. Finally, the absence of a comprehensive Japanese eQTL database limits our ability to fully validate the functional relevance of genetic variants identified in our cohort, as we relied on GTEx data obtained primarily from European descendants. This presents a major constraint, given the distinct genetic backgrounds among populations. Future studies could augment our understanding of cold hypersensitivity mechanisms by increasing validation efforts through larger epidemiological studies and integrating functional analyses of the identified genetic markers into additional experimental systems, such as in vitro and animal models.

In summary, our findings highlight a suggestive association between cold hypersensitivity, somatic burden, and genetic diversity in *KCNK2* and *TRPM2* regions. Larger sample sizes and further validation are required to confirm the role of genetic variants in cold hypersensitivity and their relationships with somatic symptoms.

### Supplementary Information

Supplementary Information.

## Data Availability

All materials, data, and protocols associated with this manuscript are available to readers upon reasonable request from the corresponding author.

## References

[1] Kimura T (1987). On the recognition and treatment of "Hiesho (Chill phobia)" in traditional kampoh medicine. Article Shoyakugaku.

[2] Bae K-H (2018). The definition and diagnosis of cold hypersensitivity in the hands and feet: Finding from the experts survey. Integr. Med. Res..

[3] Cheung, S. S. Responses of the hands and feet to cold exposure. *Temp. Multidiscipl. Biomed. J.* **2**, 105–120 (2015). 10.1080/23328940.2015.1008890 PMC4843861 27227009

[4] Stjernbrandt A, Carlsson D, Pettersson H, Liljelind I, Nilsson T, Wahlström J (2018). Cold sensitivity and associated factors: A nested case–control study performed in Northern Sweden. Int. Arch. Occup. Environ. Health.

[5] Khabbazi A (2022). Cold intolerance and associated factors: a population study. Sci. Rep..

[6] Baek Y, Jung K, Kim H, Lee S (2022). Partial sleep restriction-induced changes in stress, quality of life, and lipid metabolism in relation to cold hypersensitivity: A before-and-after intervention study. Medicine.

[7] Yoshino T (2019). Classification of patients with cold sensation by a review of systems database: A single-centre observational study. Complement. Ther. Med..

[8] Bae K-H, Go H-Y, Park K-H, Ahn I, Yoon Y, Lee S (2018). The association between cold hypersensitivity in the hands and feet and chronic disease: Results of a multicentre study. BMC Complement. Altern. Med..

[9] Kono K (2021). Vascular endothelial dysfunction and autonomic nervous hyperactivity among premenopausal women with cold-sensitivity constitution (Hiesho). Tohoku J. Exp. Med..

[10] Obermeyer Z, Samra JK, Mullainathan S (2017). Individual differences in normal body temperature: Longitudinal big data analysis of patient records. BMJ.

[11] Dodo N, Hashimoto R (2017). The effect of anxiety sensitivity on psychological and biological variables during the cold pressor test. Auton. Neurosci. Basic Clin..

[12] Zhong C-B, Leonardelli GJ (2008). Cold and lonely: Does social exclusion literally feel cold?. Psychol. Sci..

[13] Wang, Z. & Lu, Z.-Y. *A Study on the Metaphor of Social Exclusion from Embodied Cognition* (2010).

[14] Reutner L, Hansen J, Greifeneder R (2015). The cold heart: Reminders of money cause feelings of physical coldness. Soc. Psychol. Pers. Sci..

[15] Yoshino T (2013). Statistical analysis of Hie (cold sensation) and Hiesho (cold disorder) in Kampo Clinic. Evid.-Based Complement. Altern. Med..

[16] Okland TS, Gonzalez JR, Ferber AT, Mann SE (2017). Association between patient review of systems score and somatization. JAMA Otolaryngol.-Head Neck Surg..

[17] Kroenke K, Spitzer RL, Williams JB (2002). The PHQ-15: Validity of a new measure for evaluating the severity of somatic symptoms. Psychosom. Med..

[18] Gierk B (2014). The somatic symptom scale-8 (SSS-8). JAMA Intern. Med..

[19] Kondo M, Okamura Y (1987). Cold constitution: Analysis of the questionnaire. Acta Obstetr. Gynaecol. Jpn..

[20] Hur Y-M (2012). Feeling of cold hands and feet is a highly heritable phenotype. Twin Res. Hum. Genet..

[21] Key FM (2018). Human local adaptation of the TRPM8 cold receptor along a latitudinal cline. PLOS Genet..

[22] Yang L, Wang M, Wu W, Zhang L (2007). Transcriptome analysis of cold syndrome using microarray. Am. J. Chin. Med..

[23] Wang Q, Yao S (2008). Molecular basis for cold-intolerant Yang-deficient constitution of traditional Chinese medicine. Am. J. Chin. Med..

[24] Ma T, Tan C, Zhang H, Wang M, Ding W, Li S (2010). Bridging the gap between traditional Chinese medicine and systems biology: The connection of cold syndrome and NEI network. Mol. BioSyst..

[25] Arakawa K, Ishii Y, Kagawa Y (2015). Cold constitution and single nucleotide polymorphisms of β-adrenergic receptors, uncoupling protein 1 and angiotensinogen. Jpn. J. Biometeorol..

[26] Soeda M (2021). Cold pain sensitivity is associated with single-nucleotide polymorphisms of PAR2/F2RL1 and TRPM8. Mol. Pain.

[27] Hirschhorn JN, Daly MJ (2005). Genome-wide association studies for common diseases and complex traits. Nat. Rev. Genet..

[28] Uffelmann E (2021). Genome-wide association studies. Nat. Rev. Methods Primers.

[29] Wu X (2021). Relationship between conventional medicine chapters in ICD-10 and Kampo pattern diagnosis: A cross-sectional study. Front. Pharmacol..

[30] Matsudaira K (2017). Development of a Japanese version of the Somatic Symptom Scale-8: Psychometric validity and internal consistency. Gen. Hosp. Psychiatry.

[31] Purcell S (2007). PLINK: A tool set for whole-genome association and population-based linkage analyses. Am. J. Hum. Genet..

[32] Chang CC, Chow CC, Tellier LCAM, Vattikuti S, Purcell SM, Lee JJ (2015). Second-generation PLINK: Rising to the challenge of larger and richer datasets. GigaScience.

[33] Loh P-R, Palamara PF, Price AL (2016). Fast and accurate long-range phasing in a UK Biobank cohort. Nat. Genet..

[34] Loh P-R (2016). Reference-based phasing using the Haplotype Reference Consortium panel. Nat. Genet..

[35] Das S (2016). Next-generation genotype imputation service and methods. Nat. Genet..

[36] Team RC. *R: A Language and Environment for Statistical Computing*. (R Foundation for Statistical Computing, 2022).

[37] Noël J (2009). The mechano-activated K+ channels TRAAK and TREK-1 control both warm and cold perception. EMBO J..

[38] Tan C-H, Mcnaughton PA (2016). The TRPM2 ion channel is required for sensitivity to warmth. Nature.

[39] Hashimoto K, Takeuchi T, Hiiragi M, Koyama A, Nakamura Y, Hashizume M (2022). Utility and optimal cut-off point of the Somatic Symptom Scale-8 for central sensitization syndrome among outpatients with somatic symptoms and related disorders. BioPsychoSoc. Med..

[40] Guo D, Kleinstäuber M, Johnson MH, Sundram F (2019). Evaluating commonalities across medically unexplained symptoms. Int. J. Environ. Res. Public Health.

[41] Leonhart R (2018). Comparison of the factor structure of the Patient Health Questionnaire for somatic symptoms (PHQ-15) in Germany, the Netherlands, and China. A transcultural structural equation modeling (SEM) study. Front. Psychiatry.

[42] Ryder AG (2008). The cultural shaping of depression: Somatic symptoms in China, psychological symptoms in North America?. J. Abnorm. Psychol..

[43] Descoeur J (2011). Oxaliplatin-induced cold hypersensitivity is due to remodelling of ion channel expression in nociceptors. EMBO Mol. Med..

[44] Rueda-Ruzafa L, Herrera-Pérez S, Campos-Ríos A, Lamas JA (2021). Are TREK channels temperature sensors?. Front. Cell. Neurosci..

[45] Lamas JA, Rueda-Ruzafa L, Herrera-Pérez S (2019). Ion channels and thermosensitivity: TRP, TREK, or Both?. Int. J. Mol. Sci..

[46] Ujisawa T, Sasajima S, Kashio M, Tominaga M (2022). Thermal gradient ring reveals different temperature-dependent behaviors in mice lacking thermosensitive TRP channels. J. Physiol. Sci..

[47] Song K (2016). The TRPM2 channel is a hypothalamic heat sensor that limits fever and can drive hypothermia. Science.

[48] Kamm GB (2021). A synaptic temperature sensor for body cooling. Neuron.

[49] Nakao A, Matsunaga Y, Hayashida K, Takahashi N (2021). Role of oxidative stress and Ca^2+^ signaling in psychiatric disorders. Front. Cell Dev. Biol..

[50] Jang Y (2015). TRPM2, a susceptibility gene for bipolar disorder, regulates glycogen synthase kinase-3 activity in the brain. J. Neurosci..

