# Supplementary material for: Exploratory study of cold hypersensitivity in Japanese women: genetic associations and somatic symptom burden
Source: Sci Rep. 2024 Jan 22;14:1918. doi: 10.1038/s41598-024-52119-y (PMC11231259; doi:10.1038/s41598-024-52119-y)
Supplement: Supplementary file 1 — Supplementary Information. [file 41598_2024_52119_MOESM1_ESM.pdf]

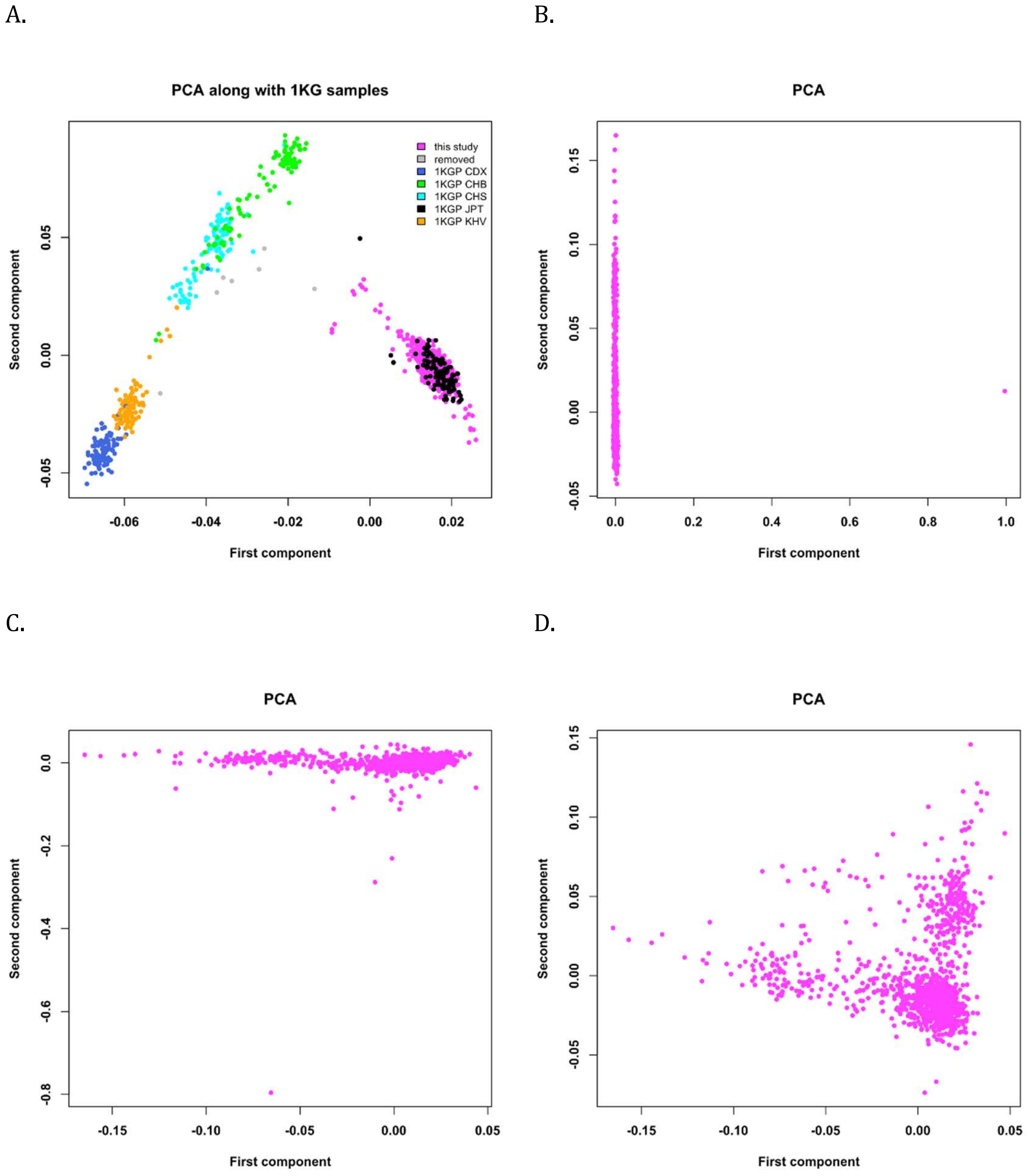

Supplementary Fig. 1 PCA plots in sample quality control (A) and SNP quality control (B-D).

A.) PCA plot within the East Asian population structure. The black points represent the 1000 Genomes Project Phase 3 Japanese population (1KGP JPT, n = 104), and grey points represent samples (n = 7) that were removed from subsequent analysis because their minimum square distance from the Japanese cluster was greater than 0.001. B-D.) Iterative PCA plots used for ethnicity identification after imputation. One sample was identified as an outlier and removed, respectively in (B) and (C). In (D), all remaining samples were confirmed as belonging to one cluster.

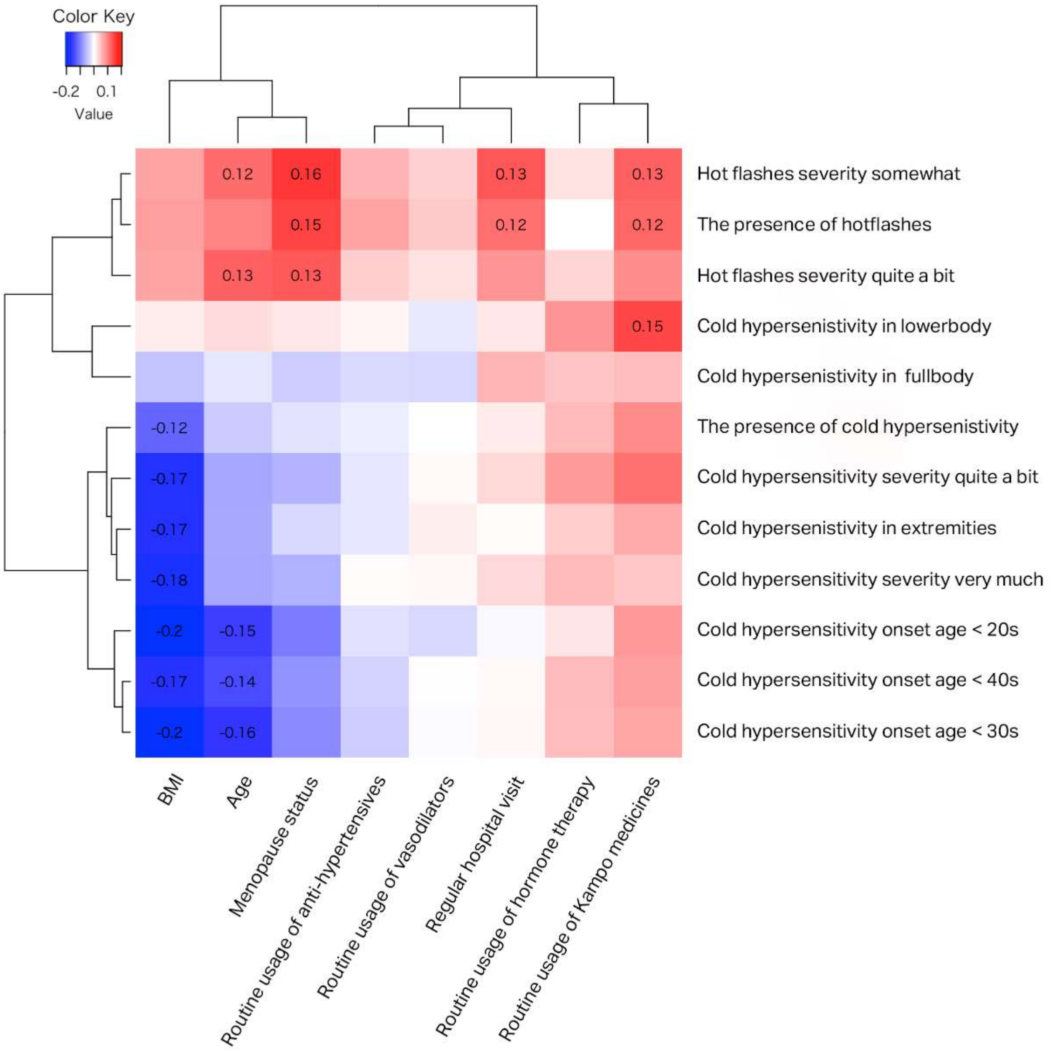

Supplementary Fig. 2 Correlation analysis between cold sensitivity and hot flashes items and demographic and medication history factors

Age, BMI, and menopausal status showed weak correlations with several cold sensitivity and hot flashes items.

1. The present of cold hypersensitivity

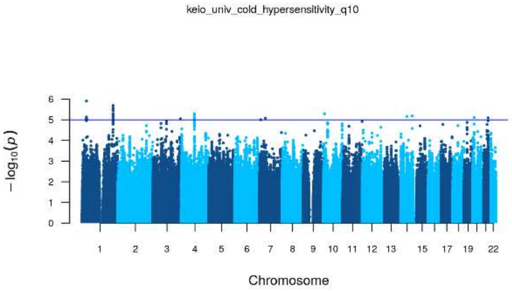

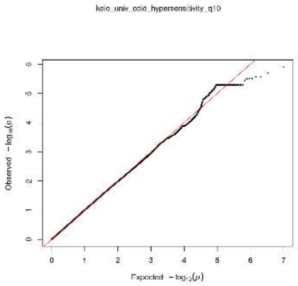

2. Cold hypersensitivity in lower body

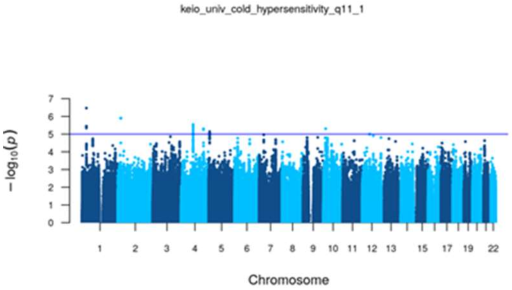

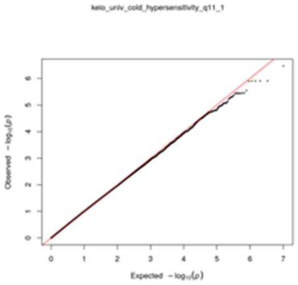

3. Cold hypersensitivity in extremities

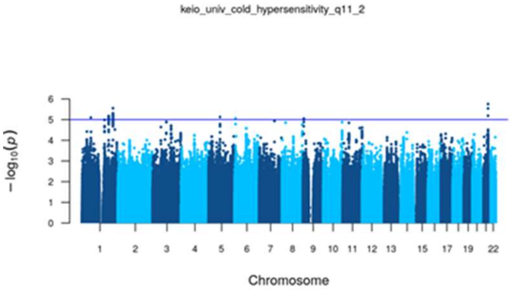

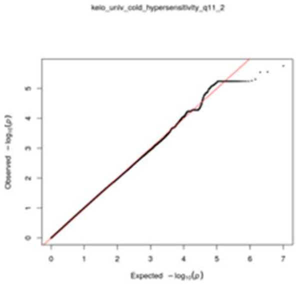

4. Cold hypersensitivity in full body

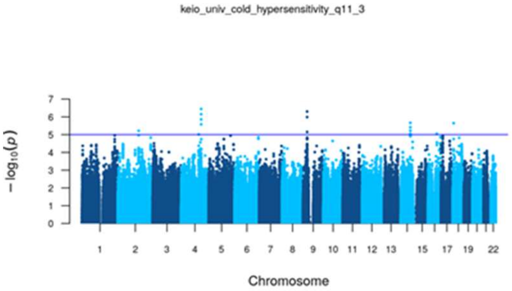

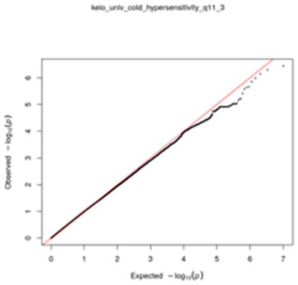

5. Cold hypersensitivity severity: quite a bit or very much

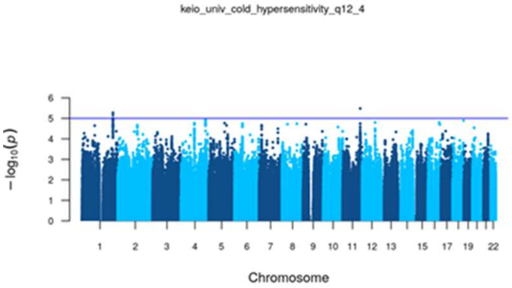

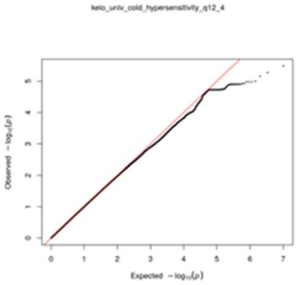

6. Cold hypersensitivity severity: very much

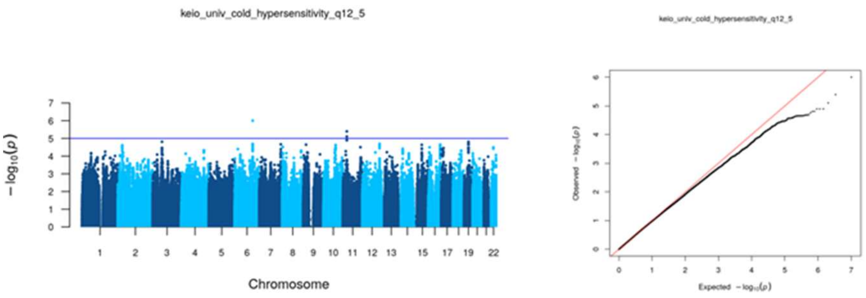

7. Cold hypersensitivity onset at < 20 years of age

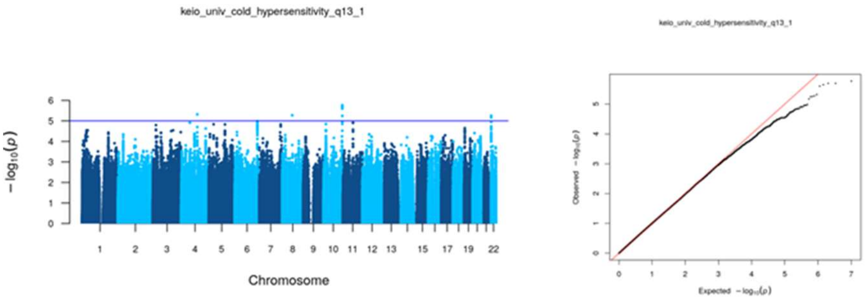

8. Cold hypersensitivity onset at < 30 years of age

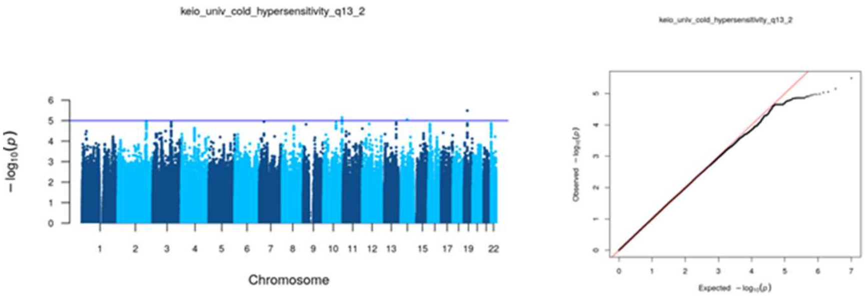

9. Cold hypersensitivity onset at < 40 years of age

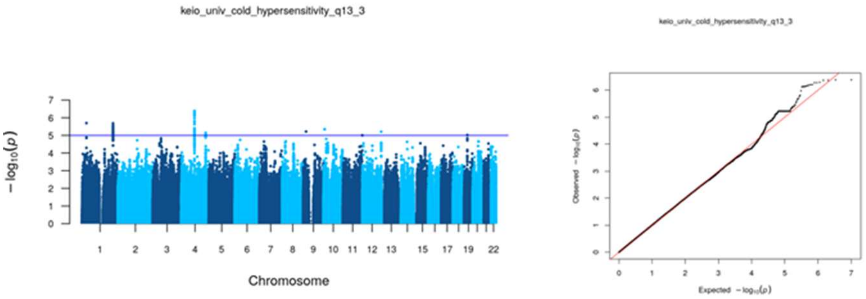

Supplementary Fig. 3 Manhattan and QQ plots of single-trait GWAS

A GWAS was performed by the main trait of "the presence of cold hypersensitivity", and sensitivity analyses were conducted by eight subordinate traits including location, severity, and onset of cold hypersensitivity stratified by decade of life. The Manhattan plot shows the -log10 transformed P-values for each SNP plotted against its chromosomal location. The blue line indicates the suggestive significance threshold 1×10^{-5}. The QQ plot displays the observed versus expected distribution of P-values after correcting for multiple testing.

A. The presence of cold hypersensitivity

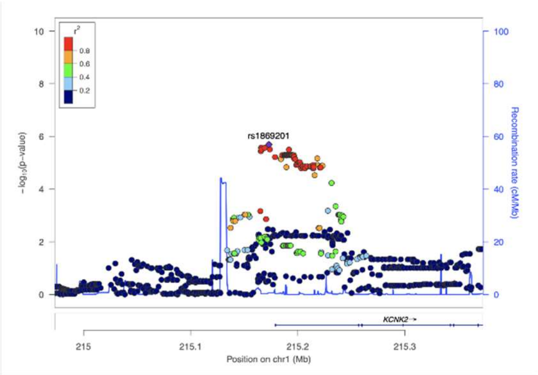

B. Cold hypersensitivity in extremities

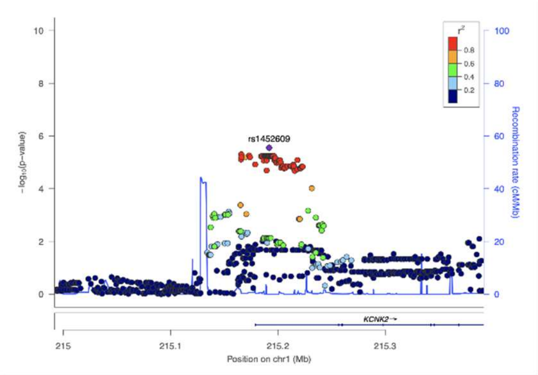

C. Cold hypersensitivity severity: quite a bit

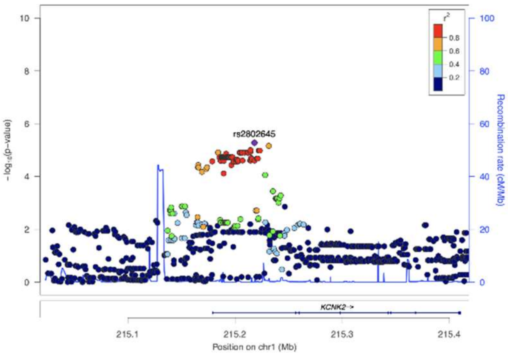

D. Cold hypersensitivity onset at < 40 years of age

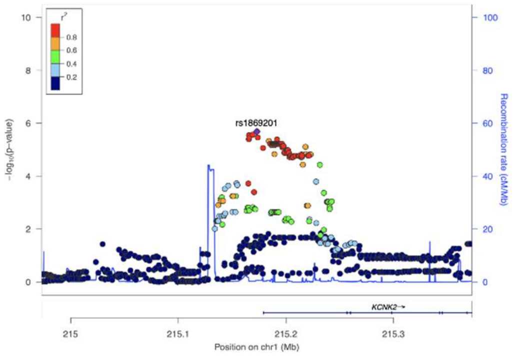

Supplementary Fig. 4 Detailed plots of the KCNK2 gene region displaying suggestive associations with cold hypersensitivity traits

Each point represents a single nucleotide polymorphism (SNP) analyzed, and its linkage disequilibrium coefficient (r^2) with the lead SNP (purple, indicated by its rs number) is indicated by color coding. LD data from the 1KGP Asian population (ASN; Mar 2014) provided by LocusZoom was used.

A. The presence of cold hypersensitivity

B. Cold hypersensitivity in extremities

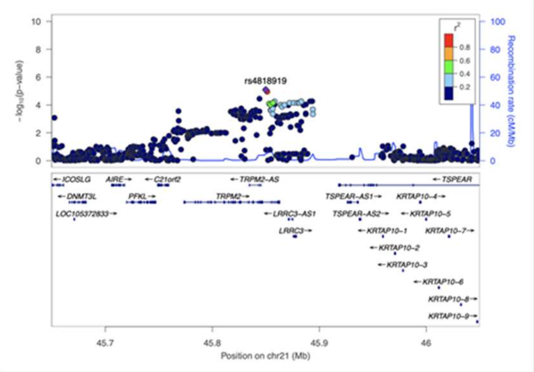

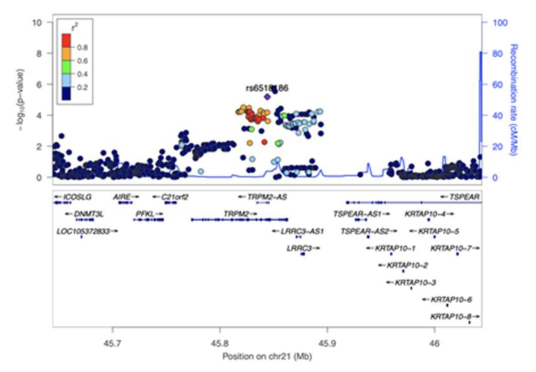

C. Cold hypersensitivity in extremities

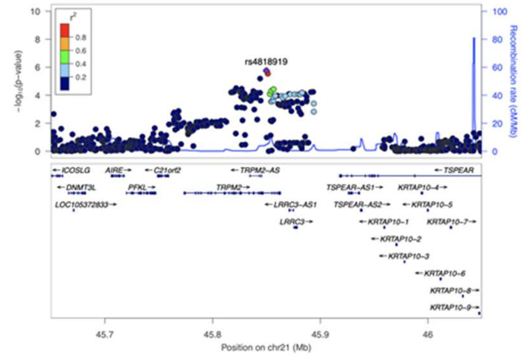

Supplementary Fig. 5 Detailed plots of the TRPM2 gene region displaying suggestive associations with cold hypersensitivity traits

Each point represents a single nucleotide polymorphism (SNP) analyzed, and its linkage disequilibrium coefficient (r^2) with the lead SNP (purple, indicated by its rs number) is indicated by color coding. LD data from the 1KGP Asian population (ASN; Mar 2014) provided by LocusZoom was used.

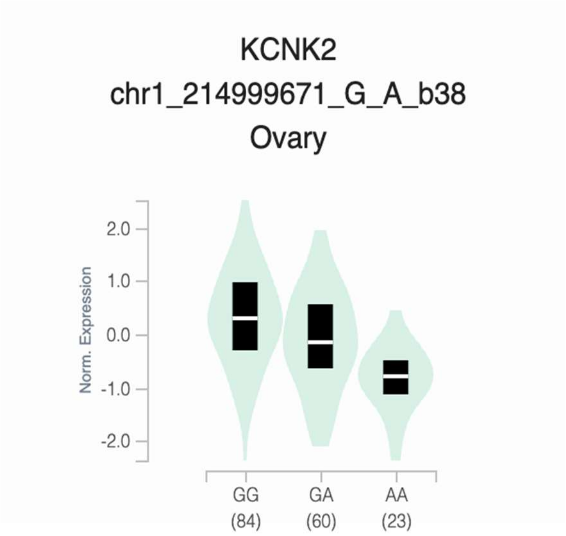

Supplementary Fig. 6 Expression quantitative trait loci (eQTL) effects of SNPs in the KCNK2 gene region.

KCNK2 expression levels stratified by rs1869201 genotype. The y-axis shows the normalized gene expression levels, and the x-axis displays the genotypes for each SNP.

A.

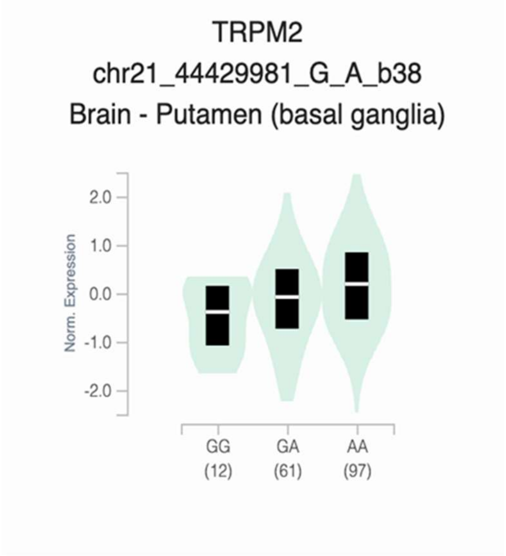

B.

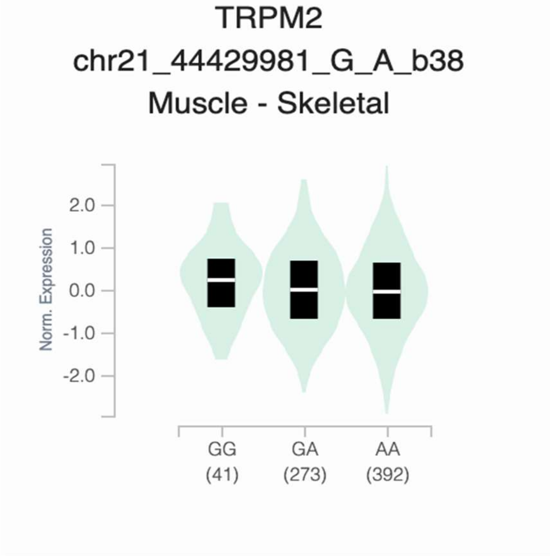

Supplementary Fig. 7 Expression quantitative trait loci (eQTL) effects of SNPs in the TRPM2 gene region

A.) TRPM2 expression levels stratified by rs4818919 genotype in brain tissue. B.) TRPM2 expression levels stratified by rs4818919 genotype in muscle-skeletal tissue. The y-axis shows the normalized gene expression levels, and the x-axis displays the genotypes for each SNP.

Supplementary Table 1 Questionnaire

Question. No	Question	Display condition	Option
**Please answer the following questions about yourself at present.**			
1	Current age	All	
2	Current height	All	
3	Current weight	All	
4	Are you currently hospitalized	All	No<br>Yes
5	Are you currently menopaused	All	No<br>Yes
6	Are you currently pregnant or possibly pregnant	who answered "no" to Q5	No<br>Yes
**Please select those that apply to you in the last few weeks.**			
7	Do you exercise, sweating lightly, for over 30 minutes a time, at least twice a week?	All	No<br>Yes
8	Do you skip breakfast more than 2-3 times per week?<br><br>*skip breakfast is defined as: (1) no breakfast, or (2) eating only nutritional supplements such as pills or nutritional drinks, or (3) eating only foods such as sweets, fruits, dairy products, and beverages.	All	No<br>Yes
9	How is your average walking speed compared to your same-age peers?	All	Slow<br>Normal<br>Fast
**Please answer the following questions regarding cold hypersensitivity or hot flashes in the last few weeks.**			
10	Do you currently feel cold hypersensitivity?	All	No<br>Yes
11	Where do you feel cold the most?	who answered "yes" to Q10	lower body<br>extremities<br>full body
12	How much do you suffer from cold hypersensitivity?	who answered	not at all<br>a little bit<br>somewhat

Question. No	Question	Display condition	Option
		"yes" to Q10	quite a bit<br>very much
13	How old were you when you first felt cold hypersensitivity?	who answered "yes" to Q10	10s<br>20s<br>30s<br>40s<br>50s<br>60s or older
14	Are you currently feeling hot flashes or heat sensation on the face?	All	No<br>Yes
15	How much do you suffer from hot flashes or heat sensation on the face?	who answered "yes" to Q14	not at all<br>a little bit<br>somewhat<br>quite a bit<br>very much
**During the past 7 days, how much have you been bothered by any of the following problems?**			
16	Stomach or bowel problems	All	not at all<br>a little bit<br>somewhat<br>quite a bit<br>very much
17	Back pain	All	not at all<br>a little bit<br>somewhat<br>quite a bit<br>very much
18	Pain in your arms, legs, or joints	All	not at all<br>a little bit<br>somewhat<br>quite a bit<br>very much
19	Headaches	All	not at all<br>a little bit<br>somewhat<br>quite a bit<br>very much
20	Chest pain or shortness of breath	All	not at all<br>a little bit<br>somewhat<br>quite a bit<br>very much
21	Dizziness	All	not at all<br>a little bit<br>somewhat

Question. No	Question	Display condition	Option
			quite a bit<br>very much
22	Feeling tired or having low energy	All	not at all<br>a little bit<br>somewhat<br>quite a bit<br>very much
23	Trouble sleeping	All	not at all<br>a little bit<br>somewhat<br>quite a bit<br>very much
**Please answer the following questions about your current medical condition(s).**			
24	Are you currently visiting the hospital regularly	All	No<br>Yes
25	Please select all of the following that applies to the disease(s) you are currently diagnosed or being treated for.<br>If there is no disease name that applies to you, please select "Other."	who answered "yes" to Q24	Heart failure, Abnormal thyroid function (hyperthyroidism, hypothyroidism, chronic thyroiditis, Hashimoto's disease), Malignant tumor, Hypopituitarism, Somatoform autonomic dysfunction (so-called dysautonomia), Arteriosclerosis obliterans, Menopausal disorders, Dysmenorrhea, Anxiety disorders, Rheumatoid arthritis, Addison's disease, Raynaud's disease, Systemic lupus erythematosus, High blood pressure, Low blood pressure, Angina pectoris, Myocardial infarction, Congenital heart disease, Valvular heart disease, Cerebrovascular disease (cerebral infarction/hemorrhage), Depression, Ulcerative colitis, Irritable bowel syndrome, Gastritis, gastric ulcer, duodenal ulcer, Pancreatitis, Reflux esophagitis, Colorectal polyp, Chronic hepatitis (hepatitis B and C), Liver cirrhosis, Fatty liver, Alcoholic liver disease, Diabetes mellitus, Chronic kidney disease, Chronic nephritis (nephrosis), Chronic bronchitis, Bronchial asthma, Emphysema, Hypercholesterolemia, Hyperuricemia (gout), Sleep apnea syndrome, Migraine headache, Uterine fibroids, Osteoarthritis, Osteoporosis, Neuralgia, Atopic dermatitis, Hemorrhoids, Urinary tract stones, Cholelithiasis, Other.
26	Please enter the name of the "Other" disease(s). If there is more than one, please list all.	who answered "Other" to Q25	

Question. No	Question	Display condition	Option
**Please describe your use of medications in the last few weeks.**			
27	Routine usage of Kampo medicines	All	No<br>Yes
28	Routine usage of medicines to reduce blood pressure	All	No<br>Yes
29	Routine usage of hormone therapy*<br><br>*Hormone replacement therapy is a treatment method to replace female hormones with oral or topical medications to treat menstrual abnormalities, menopausal disorders, etc.	All	No<br>Yes
30	Routine usage of medicines to dilate blood vessels	All	No<br>Yes

Supplementary Table 2 Summary of case and control group sizes in each GWAS

GWAS	Case number	Control number
The presence of cold hypersensitivity	599	512
Cold hypersensitivity in lower body	139	512
Cold hypersensitivity in extremities	408	512
Cold hypersensitivity in full body	52	512
Cold hypersensitivity severity: quite a bit or very much	233	512
Cold hypersensitivity severity: very much	72	512
Cold hypersensitivity onset at < 20 years of age	207	512
Cold hypersensitivity onset at < 30 years of age	373	512
Cold hypersensitivity onset at < 40 years of age	493	512

We removed the single nucleotide polymorphisms with Hardy-Weinberg equilibrium (n = 60, $P < 1.0\times10^{-6}$) or minor allele frequency < 0.05 (n = 2157426) in analyzed population and analyzed 5077326 SNPs in GWASs

Supplementary Table 3 GWAS results reaching suggestive significance level in the population without regular use of Kampo medication

Trait	Regio No	Lead SNP	CHR	BP	P change	Filtered P	P	Filtered NMISS	Filtered OR	Filtered SE	UCSC	Overlapped gene	Nearest upstream gene	Nearest downstream gene
The presence of cold hypersensitivity	1	rs750707	1	30282320	-	3.14E-06	1.24E-06	949	1.59	0.1		.	*RP4-656G21.1*	*AL645944.1*
	2	rs1869201	1	215173014	-	5.19E-06	2.04E-06	949	0.65	0.1		.	*RP11-323K10.1*	*KCNK2*
	3	rs62295776	3	191853763	N. Imp., N.S.	5.35E-05	8.91E-06	949	0.64	0.11		.	*RN7SKP222*	*FGF12*
	4	rs10016576	4	91484515	Imp.	1.98E-07	5.07E-06	949	1.65	0.1	intronic:*CCSER1* ;non-coding intronic:*CCSER1*	*CCSER1*	.	.
	5	rs74513903	7	10410139	N. Imp., N.S.	4.28E-05	9.98E-06	949	0.62	0.12		.	*U3*	*HSPA8P8*
	6	rs2656514	7	42777914	N. Imp., N.S.	0.000345	8.35E-06	949	0.69	0.1		.	*AC027269.2*	*TCP1P1*
	7	rs12761019	10	7788209	N. Imp., N.S.	0.000338	5.10E-06	949	0.58	0.15	intronic:*ITIH2*	*ITIH2*	.	.
	8	rs17098278	14	61831860	-	1.82E-06	6.85E-06	949	0.57	0.12	intronic:*PRKCH*	*PRKCH*	.	.
	9	rs10220484	14	99457631	N. Imp., N.S.	6.66E-05	6.49E-06	949	1.53	0.11		.	*AL162151.3*	*AL162151.4*
	10	rs2876278	20	12230024	-	7.22E-06	7.78E-06	949	1.56	0.1		.	*RP4-796I8.1*	*RP11-102J14.1*
	11	rs4818919	21	45849864	N. Imp., N.S.	2.29E-05	8.09E-06	949	1.77	0.13	intronic:*TRPM2* ;non-coding intronic:*TRPM2*	*TRPM2*	.	.
Cold hypersensitivity in lower body	12	rs750707	1	30282320	-	2.84E-06	3.42E-07	555	2.12	0.16		.	*RP4-656G21.1*	*AL645944.1*
	13	rs76388277	2	20706287	N. Imp., N.S.	1.55E-05	1.25E-06	555	2.52	0.21		.	*AC012065.4*	*HS1BP3*
	14	rs6819504	4	82983435	N. Imp., N.S.	2.29E-05	2.86E-06	555	1.97	0.16		.	*RASGEF1B*	*HNRNPA3P13*
	15	rs9994962	4	154877509	-	4.33E-06	5.08E-06	555	2.17	0.17		.	*AC020703.1*	*AC079298.1*
	16	rs72719006	5	7023314	-	5.30E-06	7.22E-06	555	2.9	0.23		*RP11-122F24.1*	.	.
	17	rs13376933	10	16001237	N. Imp., N.S.	1.93E-05	4.96E-06	555	0.48	0.17		.	*snoU13*	*SNORA31*
Cold hypersensitivity in extremities	18	rs556161	1	61324813	N. Imp., N.S.	0.000188	7.96E-06	795	1.48	0.11		.	*RP11-436K8.1*	*NFIA*
	19	rs10911744	1	185479240	N. Imp., N.S.	7.25E-05	6.61E-06	795	1.51	0.1		.	*CTA-277P6.1*	*GS1-204I12.1*
	20	rs1452609	1	215191743	-	2.15E-06	2.80E-06	795	0.6	0.11	intronic:*KCNK2* ;non-coding intronic:*KCNK2*	*KCNK2*	.	.
	21	rs61998228	5	79286419	N. Imp., N.S.	1.46E-05	7.46E-06	795	2.2	0.18	3utr:*AX746944* ;intronic:*MTX3* ;coding:*MTX3* :nonsyn:*R50K*	*MTX3*	.	.
	22	rs849057	6	6505406	-	7.63E-06	8.66E-06	795	0.6	0.11	non-coding intronic:*LY86-AS1*	*LY86-AS1*	.	.
	23	rs6476958	9	5280843	-	4.73E-06	8.92E-06	795	1.66	0.11		.	*INSL4*	*RLN2*
	24	rs6518186	21	45844056	N. Imp., N.S.	8.46E-05	6.49E-06	795	1.58	0.12	5upstream:*TRPM2* ;non-coding intronic:*TRPM2* ;intronic:*TRPM2*	*AP001065.2; TRPM2*	.	.
	25	rs4818919	21	45849864	N. Imp., N.S.	1.01E-05	1.76E-06	795	1.9	0.14	intronic:*TRPM2* ;non-coding intronic:*TRPM2*	*TRPM2*	.	.
Cold hypersensitivity in full body	26	rs12615626	2	145057036	N. Imp., N.S.	0.001191	6.14E-06	477	2.56	0.29	intronic:*GTDC1*	*GTDC1*	.	.
	27	rs1513696	4	122310776	N. Imp., N.S.	0.000584	9.76E-06	477	3.1	0.33		.	*QRFPR*	*AC093816.1*
	28	rs79196079	4	138948984	-	3.05E-06	3.59E-07	477	4.31	0.31	non-coding:*LINC00616*	*LINC00616*	.	.
	29	rs9777637	9	28167489	N. Imp.	9.01E-06	5.03E-07	477	4.09	0.32	intronic:*LINGO2*	*LINGO2*	.	.
	30	rs78458106	14	86055732	-	2.48E-06	2.17E-06	477	5.2	0.35	intronic:*FLRT2*	*FLRT2*	.	.
	31	rs150099343	16	60124381	N. Imp., N.S.	0.000318	9.05E-06	477	2.54	0.26		.	*RP11-430C1.1*	*RP11-457D20.1*
	32	rs6506395	18	6474590	N. Imp., N.S.	0.000476	2.28E-06	477	3.58	0.36		.	*RPL6P27*	*CTD-2124B20.2*
Cold hypersensitivity severity: quite a bit or very much	33	rs2802645	1	215218173	N. Imp., N.S.	1.04E-05	5.31E-06	638	0.57	0.13	intronic:*KCNK2* ;non-coding intronic:*KCNK2*	*KCNK2*	.	.
	34	rs12417339	11	121110311	-	8.85E-06	3.29E-06	638	2.4	0.2		.	*RP11-775A1.2*	*RPS4XP12*
Cold hypersensitivity severity: very much	35	rs9375359	6	125032336	-	4.57E-06	9.93E-07	500	2.86	0.23	intronic:*NKAIN2*	*NKAIN2*	.	.
	36	rs724202	11	26575678	N. Imp., N.S.	2.02E-05	4.02E-06	500	2.85	0.25	intronic:*ANO3*	*ANO3*	.	.

Cold hypersensitivity onset at < 20 years of age	37	rs17041448	4	111132948	N. Imp., N.S.	2.91E-05	4.80E-06	613	1.81	0.14		.	*ELOVL6*	*HSBP1P2*
	38	rs77975469	8	69854232	N. Imp., N.S.	1.47E-05	5.32E-06	613	2.5	0.21	non-coding intronic:LOC100505718	*RP11-600K15.1*	.	.
	39	rs74935603	10	130548790	-	5.99E-06	1.70E-06	613	1.91	0.14		.	*RP11-264E18.1*	*RP11-442O18.2*
	40	rs9619067	22	17807431	N. Imp., N.S.	6.04E-05	5.58E-06	613	0.54	0.15		.	*CECR3*	*CECR9*
Cold hypersensitivity onset at < 30 years of age	41	rs138993776	10	128281984	N. Imp., N.S.	6.96E-05	7.05E-06	760	0.54	0.15	intronic:*C10orf90*	*C10orf90*	.	.
	42	rs17098278	14	61831860	-	8.25E-06	8.86E-06	760	0.53	0.14	intronic:*PRKCH*	*PRKCH*	.	.
	43	rs76695906	19	21766710	N. Imp., N.S.	2.63E-05	3.25E-06	760	0.54	0.15	5upstream:*AX748435*	*RP11-678G14.3*	.	.
Cold hypersensitivity onset at < 40 years of age	44	rs750707	1	30282320	N. Imp., N.S.	1.37E-05	2.01E-06	861	1.58	0.1		.	*RP4-656G21.1*	*AL645944.1*
	45	rs1869201	1	215173014	-	4.72E-06	2.08E-06	861	0.63	0.1		.	*RP11-323K10.1*	*KCNK2*
	46	rs6532233	4	91465389	Imp.	1.04E-08	4.15E-07	861	1.81	0.1	intronic:*CCSER1* ;non-coding intronic:*CCSER1*	*CCSER1*	.	.
	47	rs1962354	4	169426659	N. Imp., N.S.	9.98E-05	7.12E-06	861	1.49	0.1	intronic:*PALLD*	*DDX60L;PALLD*	.	.
	48	rs10964259	9	19740463	N. Imp., N.S.	9.09E-05	6.07E-06	861	1.49	0.1	intronic:*SLC24A2*	*SLC24A2*	.	.
	49	rs12761019	10	7788209	N. Imp., N.S.	0.000241	4.50E-06	861	0.55	0.16	intronic:*ITIH2*	*ITIH2*	.	.
	50	rs4335563	11	134354648	N. Imp., N.S.	1.53E-05	9.92E-06	861	0.63	0.11	non-coding intronic:*LOC283177* ;5upstream:*AK095081*	*RP11-627G23.1*	.	.
	51	rs77906076	12	130746327	N. Imp., N.S.	2.89E-05	6.17E-06	861	2.44	0.21		*RP11-143E21.6*	.	.
	52	rs76695906	19	21766710	N. Imp., N.S.	8.86E-05	9.56E-06	861	0.6	0.13	5upstream:*AX748435*	*RP11-678G14.3*	.	.

CHR, chromosome; BP, base-pair GRCh37 genomic coordinate;

P change: Change in GWAS *P* values in the population without regular use of Kampo medication (Improved (Imp.), P value decreased by more than one digit; Not-improved (N. Imp.), P value increased by more than one digit; Not Improved, Not Significant (N. Imp., N.S.), P value increased by more than one digit and did not meet the suggested significance level; -, P value change is smaller than one digit)

Filtered *P*, GWAS *P*-value in the population without regular use of Kampo medication; P, GWAS *P* value in the whole population; Filtered NMISS, GWAS in the population without regular use of Kampo medicine; number of samples analyzed; Filtered OR, GWAS in the population without regular use of Kampo medicine; odds ratio (for analysis_A1 allele); Filtered SE, Standard error of odds ratio of GWAS in the population without regular use of Kampo medicine.

Supplementary Table 4 Frequency distribution of cold hypersensitivity

Question	Options	Number (fraction)	Total
Do you currently feel cold hypersensitivity?	Yes	599 (0.539)	1111
Where do you feel cold the most?	Lower body	139 (0.232)	599
	Extremities	408 (0.681)	
	General	52 (0.087)	
How much do you suffer from cold hypersensitivity	Not at all	7 (0.012)	599
	A little bit	94 (0.157)	
	Somewhat	265 (0.442)	
	Quite a bit	161 (0.269)	
	Very much	72 (0.12)	
How old were you when you first felt cold hypersensitivity?	10s	207 (0.346)	599
	20s	166 (0.277)	
	30s	120 (0.2)	
	40s	72 (0.12)	
	50s	34 (0.057)	

Supplementary Table 5 GWAS results reaching suggestive significance level

Trait	Region No	Lead SNP	CHR	BP	A1	A2	MAF	*P* value	Odds rate	SE	UCSC	Overlapped gene	Nearest upstream gene	Nearest downstream gene
The presence of cold hypersensitivity	1	rs750707	1	30282320	G	A	0.42	1.24E-06	1.56	0.09		.	*RP4-656G21.1*	*AL645944.1*
	2	rs1869201	1	215173014	G	A	0.42	2.04E-06	0.66	0.09		.	*RP11-323K10.1*	*KCNK2*
	3	rs62295776	3	191853763	A	T	0.27	8.91E-06	0.64	0.1		.	*RN7SKP222*	*FGF12*
	4	rs10016576	4	91484515	G	C	0.48	5.07E-06	1.5	0.09	intronic:*CCSER1* ;non-coding intronic:*CCSER1*	*CCSER1*	.	.
	5	rs74513903	7	10410139	T	C	0.2	9.98E-06	0.62	0.11		.	*U3*	*HSPA8P8*
	6	rs2656514	7	42777914	G	A	0.3	8.35E-06	0.65	0.1		.	*AC027269.2*	*TCP1P1*
	7	rs12761019	10	7788209	A	G	0.11	5.10E-06	0.52	0.14	intronic:*ITIH2*	*ITIH2*	.	.
	8	rs17098278	14	61831860	C	T	0.22	6.85E-06	0.61	0.11	intronic:*PRKCH*	*PRKCH*	.	.
	9	rs10220484	14	99457631	C	T	0.31	6.49E-06	1.55	0.1		.	*AL162151.3*	*AL162151.4*
	10	rs2876278	20	12230024	G	A	0.35	7.78E-06	1.51	0.09		.	*RP4-796I8.1*	*RP11-102J14.1*
	11	rs4818919	21	45849864	G	A	0.15	8.09E-06	1.73	0.12	intronic:*TRPM2* ;non-coding intronic:*TRPM2*	*TRPM2*	.	.

Cold hypersensitivity in lower body	12	rs750707	1	30282320	G	A	0.42	3.42E-07	2.12	0.15		.	*RP4-656G21.1*	*AL645944.1*
	13	rs76388277	2	20706287	C	A	0.09	1.25E-06	2.61	0.2		.	*AC012065.4*	*HS1BP3*
	14	rs6819504	4	82983435	C	T	0.33	2.86E-06	2	0.15		.	*RASGEF1B*	*HNRNPA3P13*
	15	rs9994962	4	154877509	C	A	0.31	5.08E-06	2.02	0.15		.	*AC020703.1*	*AC079298.1*
	16	rs72719006	5	7023314	C	G	0.09	7.22E-06	2.66	0.22		*RP11-122F24.1*	.	.
	17	rs13376933	10	16001237	A	C	0.36	4.96E-06	0.49	0.16		.	*snoU13*	*SNORA31*
Cold hypersensitivity in extremities	18	rs556161	1	61324813	G	A	0.45	7.96E-06	1.55	0.1		.	*RP11-436K8.1*	*NFIA*
	19	rs10911744	1	185479240	C	T	0.47	6.61E-06	1.54	0.1		.	*CTA-277P6.1*	*GS1-204I12.1*
	20	rs1452609	1	215191743	G	A	0.42	2.80E-06	0.63	0.1	intronic:*KCNK2* ;non-coding intronic:*KCNK2*	*KCNK2*	.	.
	21	rs61998228	5	79286419	T	C	0.1	7.46E-06	2.12	0.17	3utr:*AX746944* ;intronic:*MTX3* ;coding:*MTX3* :nonsyn:R50K	*MTX3*	.	.
	22	rs849057	6	6505406	C	T	0.36	8.66E-06	0.63	0.11	non-coding intronic:*LY86-AS1*	*LY86-AS1*	.	.
	23	rs6476958	9	5280843	T	G	0.47	8.92E-06	1.57	0.1		.	*INSL4*	*RLN2*

	24	rs6518186	21	45844056	C	T	0.29	6.49E-06	1.62	0.11	5upstream:*TRPM2* ;non-coding intronic:*TRPM2* ;intronic:*TRPM2*	*AP001065.2;* *TRPM2*	.	.
	25	rs4818919	21	45849864	G	A	0.15	1.76E-06	1.89	0.13	intronic:*TRPM2* ;non-coding intronic:*TRPM2*	*TRPM2*	.	.
Cold hypersensitivity in full body	26	rs12615626	2	145057036	A	G	0.15	6.14E-06	3.01	0.24	intronic:*GTDC1*	*GTDC1*	.	.
	27	rs1513696	4	122310776	C	T	0.08	9.76E-06	3.59	0.29		.	*QRFPR*	*AC093816.1*
	28	rs79196079	4	138948984	G	A	0.11	3.59E-07	4	0.27	non-coding:*LINC00616*	*LINC00616*	.	.
	29	rs9777637	9	28167489	T	A	0.17	5.03E-07	3.81	0.27	intronic:*LINGO2*	*LINGO2*	.	.
	30	rs78458106	14	86055732	T	C	0.07	2.17E-06	4.36	0.31	intronic:*FLRT2*	*FLRT2*	.	.
	31	rs150099343	16	60124381	A	G	0.2	9.05E-06	2.73	0.23		.	*RP11-430C1.1*	*RP11-457D20.1*
	32	rs6506395	18	6474590	G	A	0.05	2.28E-06	4.54	0.32		.	*RPL6P27*	*CTD-2124B20.2*
Cold hypersensitivity severity: quite a bit or very much	33	rs2802645	1	215218173	T	C	0.43	5.31E-06	0.58	0.12	intronic:*KCNK2* ;non-coding intronic:*KCNK2*	*KCNK2*	.	.
	34	rs12417339	11	121110311	A	G	0.09	3.29E-06	2.38	0.19		.	*RP11-775A1.2*	*RPS4XP12*

Cold hypersensitivity severity: very much	35	rs9375359	6	125032336	T	C	0.2	9.93E-07	2.81	0.21	intronic:*NKAIN2*	*NKAIN2*	.	.
	36	rs724202	11	26575678	G	C	0.16	4.02E-06	2.72	0.22	intronic:*ANO3*	*ANO3*	.	.
Cold hypersensitivity onset at < 20 years of age	37	rs17041448	4	111132948	G	A	0.33	4.80E-06	1.83	0.13		.	*ELOVL6*	*HSBP1P2*
	38	rs77975469	8	69854232	G	A	0.1	5.32E-06	2.43	0.19	non-coding intronic:*LOC100505718*	*RP11-600K15.1*	.	.
	39	rs74935603	10	130548790	T	A	0.34	1.70E-06	1.88	0.13		.	*RP11-264E18.1*	*RP11-442O18.2*
	40	rs9619067	22	17807431	G	A	0.31	5.58E-06	0.52	0.14		.	*CECR3*	*CECR9*
Cold hypersensitivity onset at < 30 years of age	41	rs138993776	10	128281984	C	T	0.18	7.05E-06	0.53	0.14	intronic:*C10orf90*	*C10orf90*	.	.
	42	rs17098278	14	61831860	C	T	0.22	8.86E-06	0.56	0.13	intronic:*PRKCH*	*PRKCH*	.	.
	43	rs76695906	19	21766710	A	G	0.19	3.25E-06	0.53	0.14	5upstream:*AX748435*	*RP11-678G14.3*	.	.
Cold hypersensitivity onset at < 40 years of age	44	rs750707	1	30282320	G	A	0.42	2.01E-06	1.59	0.1		.	*RP4-656G21.1*	*AL645944.1*

45	rs1869201	1	215173014	G	A	0.42	2.08E-06	0.64	0.09		.	*RP11-323K10.1*	*KCNK2*
46	rs6532233	4	91465389	G	A	0.49	4.15E-07	1.62	0.1	intronic:*CCSER1* ;non-coding intronic:*CCSER1*	*CCSER1*	.	.
47	rs1962354	4	169426659	T	A	0.48	7.12E-06	1.52	0.09	intronic:*PALLD*	*DDX60L;PALLD*	.	.
48	rs10964259	9	19740463	T	C	0.38	6.07E-06	1.54	0.09	intronic:*SLC24A2*	*SLC24A2*	.	.
49	rs12761019	10	7788209	A	G	0.11	4.50E-06	0.49	0.16	intronic:*ITIH2*	*ITIH2*	.	.
50	rs4335563	11	134354648	G	A	0.39	9.92E-06	0.65	0.1	non-coding intronic:*LOC283177* ; 5upstream: *AK095081*	*RP11-627G23.1*	.	.
51	rs77906076	12	130746327	T	C	0.07	6.17E-06	2.45	0.2		*RP11-143E21.6*	.	.
52	rs76695906	19	21766710	A	G	0.19	9.56E-06	0.58	0.12	5upstream: *AX748435*	*RP11-678G14.3*	.	.

Lead SNP, SNP with the lowest *P* value in the genomic region to which the SNP under analysis belongs; CHR, chromosome; BP, base-pair GRCh37 genomic coordinate; A1, the allele for which odds ratio is calculated (minor allele); A2, the major allele; MAF, Minor allele frequency; SE, the standard error of odds ratio; UCSC, mutation type based on SNP data of from the University of California, Santa Cruz (UCSC) Genome Browser.

Supplementary Table 6 eQTL search results of lead SNPs identified from GWASs

Trait	eGenes	SNP	BP	slope	slope_se	tissue	ALT	REF
The presence of cold hypersensitivity	*ITIH2*	rs12761019	7788209	0.41	0.08	Adipose_Subcutaneous	G	A
	KIN	rs12761019	7788209	0.21	0.04	Skin_Sun_Exposed_Lower_leg	G	A
	KIN	rs12761019	7788209	0.26	0.06	Artery_Aorta	G	A
	KIN	rs12761019	7788209	0.43	0.06	Nerve_Tibial	G	A
	ITIH2	rs12761019	7788209	0.31	0.07	Muscle_Skeletal	G	A
	KIN	rs12761019	7788209	0.27	0.06	Esophagus_Muscularis	G	A
	ITIH2	rs12761019	7788209	0.41	0.10	Esophagus_Mucosa	G	A
	KIN	rs12761019	7788209	0.34	0.06	Colon_Transverse	G	A
	KIN	rs12761019	7788209	0.26	0.05	Thyroid	G	A
	AP001062.7	rs4818919	45849864	-0.22	0.05	Colon_Transverse	A	G
	TRPM2	rs4818919	45849864	0.55	0.09	Brain_Cerebellum	A	G
	MTND5P1	rs4818919	45849864	-0.18	0.05	Adipose_Subcutaneous	A	G
	TRPM2	rs4818919	45849864	0.32	0.05	Brain_Putamen_basal_ganglia	A	G
	TRPM2	rs4818919	45849864	0.53	0.10	Brain_Cerebellar_Hemisphere	A	G
	C21orf2	rs4818919	45849864	-0.13	0.03	Whole_Blood	A	G
	C21orf2	rs4818919	45849864	-0.17	0.04	Skin_Not_Sun_Exposed_Suprapubic	A	G
	TRPM2	rs4818919	45849864	0.29	0.05	Brain_Caudate_basal_ganglia	A	G
	TRPM2	rs4818919	45849864	-0.21	0.04	Muscle_Skeletal	A	G

Trait	eGenes	SNP	BP	slope	slope_se	tissue	ALT	REF
	TRPM2	rs4818919	45849864	0.30	0.05	Brain_Nucleus_accumbens_basal_ganglia	A	G
	C21orf2	rs4818919	45849864	-0.17	0.04	Nerve_Tibial	A	G
	C21orf2	rs4818919	45849864	-0.16	0.04	Cells_Cultured_fibroblasts	A	G
	KCNK2	rs1869201	215173014	-0.50	0.09	Ovary	A	G
	GLI3	rs2656514	42777914	0.13	0.03	Nerve_Tibial	A	G
Cold hypersensitivity in lower body	*RASGEF1B*	rs6819504	82983435	-0.45	0.09	Brain_Cerebellar_Hemisphere	C	T
	LDAH	rs76388277	20706287	-0.22	0.06	Adipose_Subcutaneous	C	A
Cold hypersensitivity in extremities	*TRPM2*	rs4818919	45849864	0.53	0.10	Brain_Cerebellar_Hemisphere	A	G
	C21orf2	rs4818919	45849864	-0.16	0.04	Cells_Cultured_fibroblasts	A	G
	TRPM2	rs4818919	45849864	0.30	0.05	Brain_Nucleus_accumbens_basal_ganglia	A	G
	C21orf2	rs4818919	45849864	-0.17	0.04	Skin_Not_Sun_Exposed_Suprapubic	A	G
	AP001062.7	rs4818919	45849864	-0.22	0.05	Colon_Transverse	A	G
	TRPM2	rs4818919	45849864	0.55	0.09	Brain_Cerebellum	A	G
	TRPM2	rs4818919	45849864	0.32	0.05	Brain_Putamen_basal_ganglia	A	G
	C21orf2	rs4818919	45849864	-0.17	0.04	Nerve_Tibial	A	G
	TRPM2	rs4818919	45849864	-0.21	0.04	Muscle_Skeletal	A	G
	MTND5P1	rs4818919	45849864	-0.18	0.05	Adipose_Subcutaneous	A	G
	TRPM2	rs4818919	45849864	0.29	0.05	Brain_Caudate_basal_ganglia	A	G

Trait	eGenes	SNP	BP	slope	slope_se	tissue	ALT	REF
	C21orf2	rs4818919	45849864	-0.13	0.03	Whole_Blood	A	G
	LRRC3	rs6518186	45844056	-0.16	0.03	Skin_Sun_Exposed_Lower_leg	T	C
	MTND5P1	rs6518186	45844056	-0.20	0.04	Adipose_Subcutaneous	T	C
	LRRC3	rs6518186	45844056	-0.15	0.03	Breast_Mammary_Tissue	T	C
	MTND5P1	rs6518186	45844056	-0.15	0.03	Nerve_Tibial	T	C
	C21orf2	rs6518186	45844056	-0.12	0.03	Whole_Blood	T	C
	C21orf2	rs6518186	45844056	-0.21	0.05	Breast_Mammary_Tissue	T	C
	C21orf2	rs6518186	45844056	-0.18	0.04	Adipose_Subcutaneous	T	C
	LRRC3	rs6518186	45844056	-0.23	0.04	Artery_Tibial	T	C
	C21orf2	rs6518186	45844056	-0.17	0.04	Esophagus_Mucosa	T	C
	C21orf2	rs6518186	45844056	-0.17	0.04	Esophagus_Muscularis	T	C
	TRPM2	rs6518186	45844056	0.30	0.05	Brain_Nucleus_accumbens_basal_ganglia	T	C
	C21orf2	rs6518186	45844056	-0.17	0.04	Skin_Not_Sun_Exposed_Suprapubic	T	C
	TRPM2	rs6518186	45844056	0.55	0.10	Brain_Cerebellar_Hemisphere	T	C
	LRRC3	rs6518186	45844056	-0.23	0.04	Thyroid	T	C
	TRPM2	rs6518186	45844056	0.59	0.09	Brain_Cerebellum	T	C
	LRRC3	rs6518186	45844056	-0.18	0.03	Nerve_Tibial	T	C
	TRPM2	rs6518186	45844056	0.29	0.05	Brain_Putamen_basal_ganglia	T	C
	C21orf2	rs6518186	45844056	-0.18	0.04	Nerve_Tibial	T	C

Trait	eGenes	SNP	BP	slope	slope_se	tissue	ALT	REF
	C21orf2	rs6518186	45844056	-0.28	0.05	Colon_Sigmoid	T	C
	PFKL	rs6518186	45844056	0.11	0.03	Esophagus_Mucosa	T	C
	C21orf2	rs6518186	45844056	-0.18	0.05	Thyroid	T	C
	LRRC3	rs6518186	45844056	-0.57	0.12	Uterus	T	C
	TRPM2	rs6518186	45844056	-0.18	0.04	Muscle_Skeletal	T	C
	LRRC3	rs6518186	45844056	-0.19	0.04	Cells_Cultured_fibroblasts	T	C
	AP001062.7	rs6518186	45844056	-0.17	0.04	Skin_Not_Sun_Exposed_Suprapubic	T	C
	TRPM2	rs6518186	45844056	0.30	0.05	Brain_Caudate_basal_ganglia	T	C
	LRRC3	rs6518186	45844056	-0.15	0.04	Esophagus_Muscularis	T	C
	TRPM2	rs6518186	45844056	-0.18	0.03	Whole_Blood	T	C
	LRRC3	rs6518186	45844056	-0.21	0.04	Adipose_Visceral_Omentum	T	C
	MTX3	rs61998228	79286419	-0.55	0.10	Esophagus_Muscularis	T	C
	MTX3	rs61998228	79286419	-0.35	0.08	Colon_Transverse	T	C
	MTX3	rs61998228	79286419	-0.35	0.07	Artery_Tibial	T	C
	MTX3	rs61998228	79286419	-0.46	0.08	Thyroid	T	C
	MTX3	rs61998228	79286419	-0.41	0.09	Testis	T	C
	CTC-512J14.5	rs61998228	79286419	1.78	0.40	Prostate	T	C
	MTX3	rs61998228	79286419	-0.61	0.12	Heart_Atrial_Appendage	T	C
	IVNS1ABP	rs10911744	185479240	0.12	0.03	Adipose_Subcutaneous	C	T

Trait	eGenes	SNP	BP	slope	slope_se	tissue	ALT	REF
	LY86	rs849057	6505406	0.19	0.03	Lung	T	C
	LY86	rs849057	6505406	0.18	0.04	Colon_Transverse	T	C
	LY86	rs849057	6505406	0.15	0.04	Esophagus_Muscularis	T	C
	KCNK2	rs1452609	215191743	-0.67	0.08	Ovary	A	G
	RLN2	rs6476958	5280843	0.19	0.05	Testis	T	G
Cold hypersensitivity in full body	*L3MBTL4*	rs6506395	6474590	-0.17	0.04	Esophagus_Mucosa	G	A
	L3MBTL4	rs6506395	6474590	-0.18	0.03	Skin_Sun_Exposed_Lower_leg	G	A
	L3MBTL4	rs6506395	6474590	-0.15	0.03	Skin_Not_Sun_Exposed_Suprapubic	G	A
	QRFPR	rs1513696	122310776	-0.20	0.05	Esophagus_Mucosa	C	T
	QRFPR	rs1513696	122310776	-0.48	0.08	Testis	C	T
	RP11-364P2.2	rs1513696	122310776	-0.22	0.05	Testis	C	T
Cold hypersensitivity severity: very much	*MUC15*	rs724202	26575678	-0.10	0.02	Esophagus_Mucosa	C	G
	MUC15	rs724202	26575678	-0.42	0.07	Stomach	C	G
	ANO3	rs724202	26575678	0.54	0.07	Pancreas	C	G
Cold hypersensitivity onset at < 20 years of age	*GAR1*	rs17041448	111132948	-0.11	0.03	Muscle_Skeletal	G	A
Cold hypersensitivity onset at < 40 years of age	*ITIH2*	rs12761019	7788209	0.41	0.08	Adipose_Subcutaneous	G	A

Trait	eGenes	SNP	BP	slope	slope_se	tissue	ALT	REF
	KIN	rs12761019	7788209	0.43	0.06	Nerve_Tibial	G	A
	KIN	rs12761019	7788209	0.26	0.05	Thyroid	G	A
	ITIH2	rs12761019	7788209	0.41	0.10	Esophagus_Mucosa	G	A
	KIN	rs12761019	7788209	0.27	0.06	Esophagus_Muscularis	G	A
	KIN	rs12761019	7788209	0.21	0.04	Skin_Sun_Exposed_Lower_leg	G	A
	KIN	rs12761019	7788209	0.34	0.06	Colon_Transverse	G	A
	KIN	rs12761019	7788209	0.26	0.06	Artery_Aorta	G	A
	ITIH2	rs12761019	7788209	0.31	0.07	Muscle_Skeletal	G	A
	KCNK2	rs1869201	215173014	-0.50	0.09	Ovary	A	G
	DDX60L	rs1962354	169426659	0.09	0.02	Whole_Blood	A	T

BP: Genomic location specified by base-pair coordinates (GRCh37).

Slope: Represents the regression slope for the alternative allele, as obtained from GTEx results; slope_se: Standard error of the regression slope, according to GTEx results; tissue: Specifies the tissue, as detailed in the GTEx results; ALT, Alternative allele; REF, Reference all
